# Effects of combining two techniques of non-invasive brain stimulation in subacute stroke patients: a pilot study

**DOI:** 10.1186/s12883-022-02607-3

**Published:** 2022-03-17

**Authors:** Sutthikit Pipatsrisawat, Jakkrit Klaphajone, Kittipong Kitisak, Somporn Sungkarat, Pakorn Wivatvongvana

**Affiliations:** 1 Department of Rehabilitation Medicine, Faculty of Medicine, Chiang Mai University, Chiang Mai, 50200 Thailand; 2 Department of Physical Therapy, Faculty of Associated Medical Sciences, Chiang Mai University, Chiang Mai, 50200 Thailand

**Keywords:** Stroke recovery, Fugl-Meyer upper extremity motor score (FMA-UE), Wolf motor function test (WMFT), Repetitive transcranial magnetic stimulation (rTMS), Transcranial direct current stimulation (tDCS), Non-invasive brain stimulation (NIBS)

## Abstract

**Background:**

Strokes have recently become a leading cause of disability among Thai people. Non-invasive brain stimulation (NIBS) seems to give promising results in stroke recovery when combined with standard rehabilitation programs.

**Objective:**

To evaluate the combined effect of low-frequency repetitive transcranial magnetic stimulation (rTMS) and cathodal transcranial direct current stimulation (tDCS) over the non-lesional primary motor cortex on upper limb motor recovery in patients with subacute stroke. No reports of a combination of these two techniques of NIBS were found in the relevant literature.

**Methods:**

This pilot study was a double-blinded, randomized controlled trial of ten patients with subacute stroke admitted to the Rehabilitation Medicine Inpatient Unit, Maharaj Nakorn Chiang Mai Hospital, Chiang Mai University. They were randomized into two groups: five in an active and five in a sham intervention group. Fugl-Meyer’s upper extremity motor score (FMA-UE) and Wolf Motor Function Test (WMFT) were used to assess motor recovery at baseline, immediately, and 1 week after stimulation.

**Results:**

A two-way repeated ANOVA (mixed design) showed a significant improvement in FMA-UE scores in the active intervention group both immediately and 1 week after stimulation in comparison to the baseline, [time, *F* (2, 16) = 27.44, *p* < 0.001, time x group interaction, *F* (2, 16) = 13.29, *p* < 0.001]. Despite no statistical significance, a trend toward higher WMFT scores was shown in the active intervention group.

**Conclusions:**

A single session of low-frequency rTMS and cathodal tDCS over the non-lesional primary motor cortex may enhance upper limb motor recovery in patients with subacute stroke.

**Supplementary Information:**

The online version contains supplementary material available at 10.1186/s12883-022-02607-3.

## Background

Strokes (cerebrovascular disease) have recently become a major public health problem in many countries, including Thailand, with incidence climbing steadily every year. In 2017, public health statistics revealed that stroke was the second leading cause of disability-adjusted life year loss in both males and females in the Thai population [1]. This is in line with the 2017 WHO report of four major non-communicable diseases, the leading cause of global death being stroke [2]. Strokes also yielded the highest prevalence of disability among stroke survivors in the US [2, 3]. Those with disabilities in this group had disorders of mobility and limb movement, which led to difficulties in major life areas [3]. Overall incapacities put a burden on patients, families, and society as a whole, with a large proportion of national resources used in patient care [4, 5].

In Thailand, the best stroke rehabilitation program includes daily intensive rehabilitation training, which comprises conventional physical therapy (PT) and occupational therapy (OT), during admission to tertiary care hospitals. However, the usual length of stay is approximately 1 month, including comprehensive training to maximize functions reflected in the Barthel Index (BI) score. The majority of our patients, including outpatients and patients who receive “outreach” home programs, were prescribed less intensive training, which inevitably required a prolonged treatment process that achieved lower levels of improved independence [6–8]. Therefore, an upcoming trend in developed countries toward Non-Invasive Brain Stimulation (NIBS), which consists of repetitive Transcranial Magnetic Stimulation (rTMS) and transcranial Direct Current Stimulation (tDCS), is emerging as a powerful adjuvant therapy to promote better outcomes in stroke rehabilitation. Recently, NIBS has yielded promising results in terms of competency, with a shorter recovery time necessary for patients returning to their activities of daily living [9–13]. We attempted to incorporate these combination techniques into routine programs to improve the functional outcomes for patients who have suffered a stroke.

In most studies, the application of a single intervention of NIBS was performed, using either rTMS or tDCS in one phase before or during a conventional PT/OT program. The stimulation frequency recommended was five consecutive days per week with an arbitrary two-to-six-week duration [14–17]. This eventually necessitated a two to three-fold increase in the cost of the stimulation for each patient in standard rehabilitation training, which could not be reimbursed to a patient by the Thailand diagnosis-related group system. If we applied this setting to outpatient clinics, patients would need to visit the hospital daily, which is inconvenient for most patients and increases the cost of transportation. An additional problem is associated with the limited number of personnel who are able to operate the session – only trained physicians are allowed to use the NIBS machine in Thailand.

There have been few reports of the use of combined techniques of NIBS or stimulations for more than one phase during rehabilitation training. We intend to explore the combination of rTMS and tDCS at different time frames regarding safety. From our perspective, the long-term effect of NIBS would be induced by inhibitory long-term depression (LTD) [18, 19] on the contralesional side, restoring a balance between the two hemispheres [14–17], eventually enhancing changes in neural plasticity from skilled motor learning [20–22] on the ipsilesional side of a conventional rehabilitation program. From these techniques, we believed that brain stimulation using two techniques on the same day in conjunction with rehabilitation training would increase the benefit in motor learning in a non-homeostatic plasticity fashion. The results from this study may provide new knowledge and diminish the overall cost of stroke rehabilitation when using adjuvant combined NIBS.

## Methods

### Participants

We recruited ten subacute stroke patients who gave written, informed consent to participate in this study after having their stroke confirmed by CT (Computed Tomography) or MRI (Magnetic Resonance Imaging) at the Rehabilitation Medicine Inpatient Unit at Maharaj Nakorn Chiang Mai Hospital. Inclusion criteria were Thai nationals aged between 20 and 80 years, a stroke diagnosis within 3 months, and the ability to follow instructions in two consecutive steps [14, 15, 17]. Exclusion criteria were people with a history of recurrent stroke, a history of other non-cerebrovascular diseases (for example, brain tumors and brain injury), unstable medical conditions such as uncontrolled arrhythmia, acute coronary syndrome, congestive heart failure, end-stage renal disease, pneumonia, and urinary tract infection, use of a neurostimulator such as a vagal nerve or deep brain stimulator, having a pacemaker, metal in the skull or ear, a history of epilepsy, having a seizure within 1 year, history of substance abuse, pregnancy, and previous treatment with rTMS or tDCS [23, 24]. Patients were discontinued from the study if there were deliberate cancellations or if they experienced serious adverse effects such as seizures during participation [23, 24] (Data shown in Table 1).

**Table 1:** Clinical characteristics of participating patients at baseline

	Active	Sham	***P***-value
**Number of patients**	5	5	
Age*	58.8 (5.9)	59.2 (17.5)	0.97
**Time** since stroke (days)*	35.2 (21.7)	47.4 (25.0)	0.43
**Gender** - male:female	1:4	1:4	1.00^a^
**Type of stroke**			
- Ischemic stroke	4	4	1.00 ^a^
- Hemorrhagic stroke	1	1	
**Side of brain lesion**			
- Left	3	3	1.00 ^a^
- Right	2	2	
**Education**			
- Primary school	2	2	0.34^b^
- Secondary school	2	0	
- Vocational certificate	1	2	
- Higher education	0	1	
MRC-UE*	2.80 (1.09)	2.20 (2.05)	0.58
MRC-LE*	1.20 (1.30)	2.00 (1.87)	0.45
BS-ARM*	3.60 (0.89)	3.20 (1.09)	0.54
BS-HAND*	2.80 (1.30)	3.00 (1.00)	0.79
FMA-UE*	23.40 (13.47)	21.40 (12.68)	0.81
WMFT-FAS*	28.40 (11.72)	28.20 (15.01)	0.98
WMFT-TIME*	87.69 (20.71)	89.51 (29.76)	0.91

*MRC-UE* Medical Research Council scale of upper extremity, *MRC-LE* Medical Research Council scale of lower extremity, *FMA-UE* Fugl-Meyer upper extremity motor score, *WMFT-FAS* Wolf motor function test - Functional ability, *WMFT-TIME* Wolf motor function test-Performance time

^a^*P*-value by Fisher’s Exact test

^b^*P*-value by Chi-squared test

*Mean (standard deviation); *p*-value by Independence t-test

### Clinical neurobehavioral testing

To assess neurobehaviour, case report forms were completed for each patient, which included the following data: age, gender, type of stroke, time after stroke, education, Medical Research Council (MRC) scale of the upper extremity (MRC-UE) and lower extremity (MRC-LE), Brunnstrom stage of the arm (BS-ARM), Brunnstrom stage of the hand (BS-HAND), and Fugl-Meyer upper extremity motor score (FMA-UE) [25]. In addition, a 33-item evaluation of the upper arm function was carried out, assessed on a scale ranging from 0 to 2, with a maximum score of 66 points. The Wolf motor function tests (WMFT) [26] were also used. The Wolf Motor Function Test-Functional Ability Scale (WMFT-FAS) for the evaluation of the hands and arms during 15 activities, with scores ranging from 0 to 5, with a maximum cumulative score of 75, and the Wolf Motor Function Test-Performance Time (WMFT-TIME), the seconds for each activity being recorded. If a patient was unable to complete any activity within 120 s, a time of 120 s was recorded. However, in this study, the fifteenth activity, “to lift the basket,” was not applicable as it required a patient to perform in a standing position, and this would be problematic for patients with poor stability.

### Experimental design

This study was a double-blind, randomized controlled trial. All patients were recruited from the Rehabilitation Medicine In-patient Unit at Maharaj Nakorn Chiang Mai Hospital and completed informed consent forms before participating in the research program. Following screening with the inclusion and exclusion criteria, eligible patients were enrolled in the study and allocated into two groups by a trained physiatrist using computer-generated randomization. Assignments were kept unseen in brown concealed envelopes for both the experimental group (active rTMS and tDCS) and the control group (sham rTMS and tDCS). Baseline data for general characteristics, MRC-UE, MRC-LE, BS-ARM, BS-HAND, FMA-UE, and WMFT were collected by a trained physiatrist and PT before the intervention. The same PT performed the FMA-UE and WMFT immediately after completion of standard treatment and after 1 week of stimulation. Data were collected for statistical analysis by a blinded statistician who only was aware of the number of patients in each group but did not know the intervention provided to each group. In terms of blinding patients, all patients were naive to NIBS and sham conditions were the best methods available. In rTMS, the scalp contact with the coil and operating noises resembled all aspects for both groups, TDCS is equipped with a sham option [14, 15]. The room for rTMS was locked at all times during stimulation. In the case of the statisticians, the number of the group was only revealed at the end of the stimulation period (See CONSORT flow diagram).

### Stimulation parameters

The TMS machine used was a MagPro® R30 with Option, manufactured by MagVenture® A / S Lucernemarken, 15 DK-3520, Farum, Denmark. The tDCS device used was an HDCstim® from Newronika s.r.l. via Dante 4. 2012, Milan, Italy.

During rTMS sessions, another trained physiatrist, certified for NIBS, found the resting motor threshold (RMT) in the contra-lesional hemisphere. RMT indicates the minimum intensity of the stimulation that produces 3 out of 5 (> 50% successive trail) of 50 μV motor evoked potentials (MEPs) measured by the surface EMG at the contralateral abductor pollicis brevis muscle (APB). The RMT was used to calculate the optimal parameters for rTMS stimulation for each patient. The area where the RMT was obtained was used to position the coil for rTMS. In tDCS, the cathode is placed in the RMT area for rTMS, whereas the anode is placed in the contralateral supraorbital region. The stimulation process was blinded to all investigators except the operator.

The experimental group was stimulated with rTMS at 1 Hz and 100% RMT for 20 min via a figure-of-eight coil placed tangentially to the scalp. Although there was no consensus on these stimulation parameters [14–16], we applied 1 Hz as it was the lowest frequency allowed by the machine and 100% of RMT was set in line with observed activity from the EMG monitor. A total of 1200 pulses were applied in 20 min as this was the duration for which the patient could sit comfortably to avoid possible side effects [23, 27].

Immediately after the rTMS session, the cathodal tDCS was then performed with a 2-mA stimulation intensity for a 20-min duration. We selected intensity of 2 mA as this was most commonly used in earlier studies [14, 28]. Other studies have reported that some patients might experience transient minor side effects [29], i.e., a tingling sensation during electrical stimulation. Therefore, while being stimulated with the tDCS, patients were given standard occupational therapy for the next 45 min after the tDCS stimulation started [30].

The sham group did not receive either active rTMS or tDCS. In the sham rTMS, a figure-of-eight coil was placed at a perpendicular (90 degrees) angle to the scalp with both wings touching the scalp [14, 15]. The stimulation switch was turned on to produce a noise similar to the one in the active intervention group. In the sham tDCS, the current intensity was set to ramp up in the first 30 s of the stimulation and then stop [14]. After that, the electrodes were left on the scalp for 20 min during the standard OT program, the same as in the active intervention group.

The same physiatrist who operated the NIBS assessed adverse reactions and recorded these on the evaluation form for adverse reactions during and after rTMS and tDCS stimulation [23, 27, 31].

### Statistical analysis

For statistical analysis, SPSS version 25.0 was used. To analyze demographic data, descriptive statistics were used to express frequency, percentage, mean, and standard deviation. MRC-UE, MRC-LE, BS-ARM, BS-HAND, FMA-UE, and WMFT were tested for differences in general characteristics using independent t-tests for parametric data and Chi-square and Fisher’s Exact tests for nonparametric data at baseline. The Shapiro-Wilk test was used to determine the normality of the data distribution. Comparisons between experimental and control groups of MA-UE, WMFT-FAS, and WMFT-TIME were made using a 2-way repeated ANOVA (mixed design) before, immediately after, and 1 week after stimulation. The group was specified as a between-subject component, and time was specified as an intra-subject factor. The difference between groups at specific time points was tested using the Bonferroni procedure (multiple comparisons). Statistical significance was set at a *p*-value of 0.05.

## Results

All 16 patients with subacute stroke were recruited at the Rehabilitation Medicine In-patient Unit, Maharaj Nakorn Chiang Mai Hospital, six of whom were excluded because they did not meet the inclusion/exclusion criteria. The remaining ten participants were randomly divided into two groups: an experimental (active) group of five and control (sham) group of five. No one withdrew from the study. There were no statistical differences between the two groups in the general data, MRC-UE, MRC-LE, BS-ARM, BS-HAND, FMA-UE, or WMFT before participation in the research process (shown in Table 1).

Data distribution assessed using the Shapiro-Wilk test showed that the averages of FMA-UE, WMFT-FAS, and WMFT-TIME at all three-time assessments were within a normal distribution (as shown in Table 2), where further analysis with parametric tests was chosen.

**Table 2:** Distribution of the normalization test

Type of assessment	Shapiro-Wilk test	
	Active	Sham
**FMA-UE before stimulation**	0.10	0.13
**FMA-UE immediately after stimulation**	0.10	0.12
**FMA-UE 1 week after stimulation**	0.09	0.06
**WMFT-FAS before stimulation**	0.07	0.32
**WMFT-FAS immediately after stimulation**	0.25	0.34
**WMFT-FAS 1 week after stimulation**	0.31	0.28
**WMFT-TIME before stimulation**	0.87	0.11
**WMFT-TIME immediately after stimulation**	0.29	0.07
**WMFT-TIME 1 week after stimulation**	0.26	0.07

*FMA-UE* Fugl-Meyer upper extremity motor score, *WMFT-FAS* Wolf motor function test-Functional ability, *WMFT-TIME* Wolf motor function test-Performance time

### FMA-UE

**Fig. 1:**
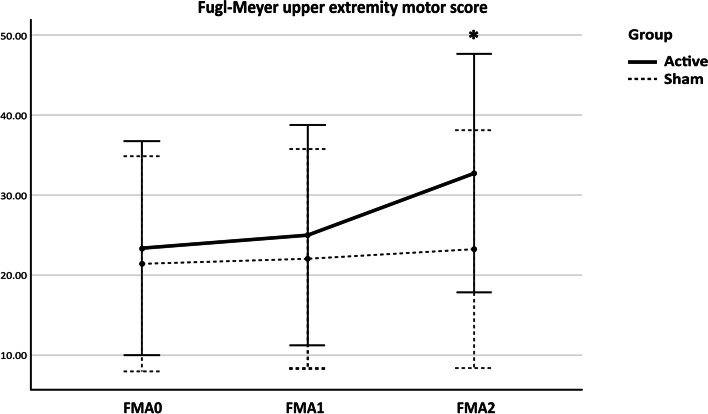
Effects of Combined NIBS/Sham on FMA-UE. FMA-UE = Fugl-Meyer upper extremity motor score; FMA0 = FMA-UE at baseline; FMA1 = FMA-UE immediately after stimulation; FMA2 = FMA-UE 1 week after stimulation; * *p* < 0.05

The 2-way mixed ANOVA was conducted. Sphericity assumed of all error variances by Mauchly’s test. Results revealed that the overall FMA-UE motor score was statistically significant across three-time points, *F* (2, 16) = 27.44, *p* < 0.001, as the estimated marginal means of FMA-UE motor scores were increasing over time in both groups, as shown in the profile plot. However, there was a significant interaction between time and experiment group, *F* (2, 16) = 13.29, *p* < .001, implying that the change in scores over time differed between the groups assigned. The data were further explored by pairwise comparisons using Bonferroni’s adjustment, and only the FM-UE of the active group had a significantly higher motor score at 1 week after stimulation, *p* < 0.001, in comparison with the baseline and immediately after stimulation, *p* = 0.001 (Fig. 1).

### WMFT-FAS and WMFT-TIME

**Fig. 2:**
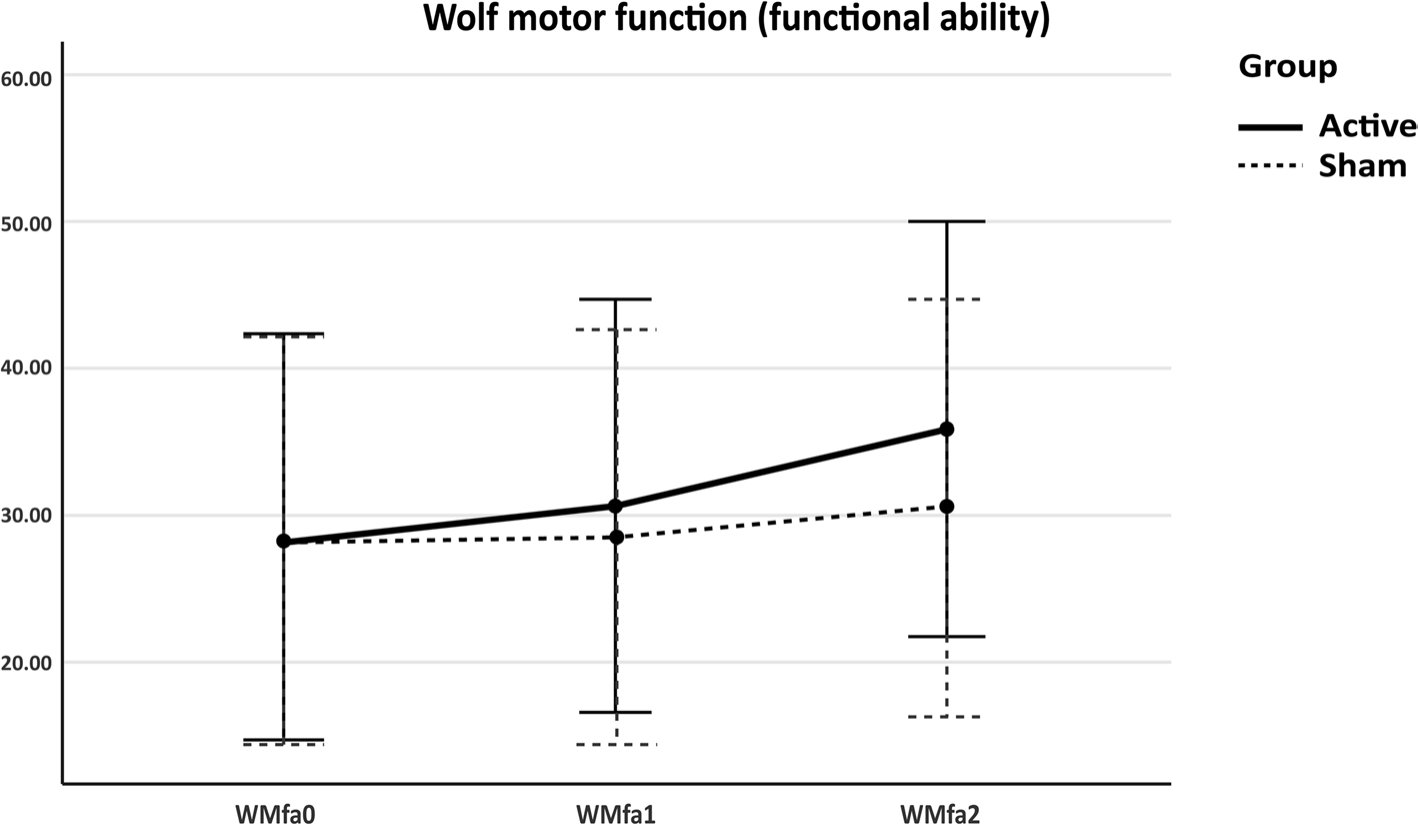
Effects of Combined NIBS/sham on WMFT-FAS. WMFT-FAS = Wolf motor function test-Functional ability; WMfa0 = WMFT-FAS at baseline; WMfa1 = WMFT-FAS immediately after stimulation; WMfa2 = WMFT-FAS 1 week after stimulation

**Fig. 3:**
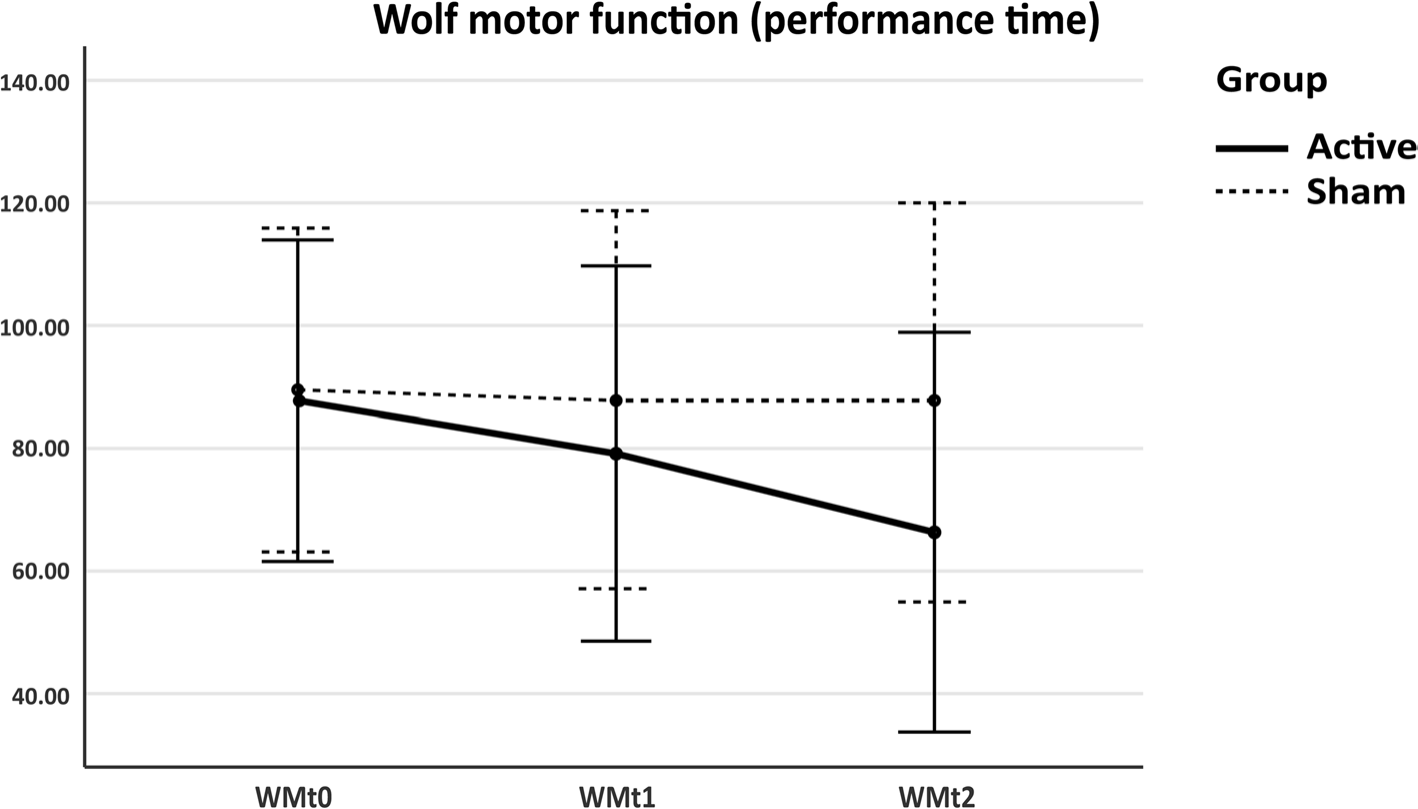
Effects of NIBS/Sham on WMFT-TIME. WMFT-TIME = Wolf motor function test-Performance time; WMt0 = WMFT-TIME at baseline; WMt1 = WMFT-TIME immediately after stimulation; WMt2 = WMFT-TIME 1 week after stimulation

The 2-way mixed ANOVA was performed. Mauchly’s test showed sphericity was assumed for WMFT-FAS but not WMFT-TIME. Therefore, we used lower-bound correction by Greenhouse-Geisser estimation. The WMFT-FAS and WMFT-TIME scores were statistically significant as regards time, *F* (2, 16) = 13.88, *p* < 0.001; *F* (1.11, 8.89) = 5.15, *p* = 0.047, respectively. However, the time and group interactions were not statistically significant, even though there was an increasing trend towards the experimental group, *F* (2, 16) = 3.37, *p* = 0.06; *F* (1.11, 8.89) = 3.80, *p* = 0.08, respectively. Both groups showed increased performance over time (Figs. 2 and 3).

### Adverse reactions of low-frequency rTMS and cathodal tDCS

Only one patient who received sham rTMS had a headache and neck pain. However, there was no adverse reaction in the active rTMS group. Two subjects experienced a burning sensation at the anode placement site during sham cathodal tDCS stimulation. Slight redness on the scalp at the anode placement was observed in three participants in real and two in sham cathodal tDCS. All adverse effects disappeared within 1 day after stimulation.

## Discussion

Few studies have reported the application of a combination of two techniques of NIBS, and either rTMS or tDCS have been chosen for designated outcomes. Almost all of the studies have shown limited results when using the majority of the resources, i.e., cumulative sessions on consecutive days and stimulation periods between 2 to 6 weeks [14–17]. Hence, we applied both techniques of NIBS (low-frequency rTMS and cathodal tDCS) to enhance upper limb function as shown on FMA-UE, WMFT-FAS, and WMFT-TIME by a single time stimulation.

One of the major adaptive CNS properties after rTMS and tDCS has been shown to be glutamatergic synaptic long-term potentiation (LTP) and long-term depression (LTD) [18], depending on the speed of postsynaptic calcium influx through N-methyl-D-aspartate (NMDA) receptors. This early stage of plasticity may last from minutes to hours. The later phase of changes in gene and protein expression may persist for hours to days [19]. Concurrent with rehabilitation training, all of this plasticity has been shown to improve motor learning [14, 15, 17].

Neural plasticity for stroke rehabilitation is based on skilled motor learning [20–22], which is to induce the sprouting of new dendrites, new synapse formation, existing synapse and axon changes, and new neurochemical production [32]. However, ideas concerning the appropriate time onset for specific stroke rehabilitation vary [20, 21]. The subacute stage for stroke recovery may be defined as 24 h to 6 weeks [14]. Also, by using the adjusted odds ratio of 3.5 increase in the likelihood of achieving the modified functional independence measure stage with a disability onset of fewer than 8 weeks [33], we used this period during the subacute stage as the optimal onset of treatment for the achievement of motor recovery.

Based on the regulation of the balance between the two hemispheres, in normal conditions, interhemispheric inhibition helps to regulate interhemispheric balance in the brain, and patients with stroke suffer from this inhibition, leading to an imbalance between the hemispheres. The non-lesional hemisphere tries to compensate by transmitting more nerve impulses to the lesional side. This results in reduced neuronal activities in the lesional hemisphere itself. In other words, motor weakness is more extensive than what is apparent from the pathological lesion [34, 35]. The principle of NIBS that affects the neurons is either to suppress the cortical excitability with low-frequency rTMS and cathodal tDCS or to increase the cortical excitability with high-frequency rTMS and anodal tDCS [14, 36].

Based on the above principles, either method has resulted in lesional hemisphere recovery [11, 35, 36]. Several studies have documented the use of low-frequency rTMS or cathodal tDCS in the non-lesional primary motor cortex and high-frequency rTMS or anodal tDCS in the lesional primary motor cortex. Both techniques have been shown to improve upper limb motor function in both the early and chronic stages of stroke patients. There has been no exact conclusion regarding the proper parameters for NIBS. However, the recommendation was made for the treatment of patients with hyperacute or early phase for low-frequency rTMS and cathodal tDCS, and chronic phase for high-frequency rTMS and anodal tDCS [14, 15, 17].

The combination of this NIBS with other rehabilitation therapy should be approached with caution [13]. Homeostatic plasticity provides a shift in the threshold for LTP and LTD induction by the Bienenstock-Cooper-Munro (BCM) principle [30, 37–39]. Timing is the most important factor between priming and test intervention, which may interfere with the designated outcome. Even though the homeostatic effect of NIBS on plasticity induced by subsequent motor learning is less consistent, to be certain, the shortest timing between priming and subsequent motor learning is the key factor in making sure that non-homeostatic plasticity will be an advantage [37].

One session of NIBS has been shown to yield only short-term results for minutes to hours [40–44]. To receive a longer cumulative effect, NIBS needs to be repeated on consecutive days [14–17]. This raises the question of whether homeostatic plasticity would take the place of another stimulation on a consecutive day or even with a shorter separate session on the same day, i.e., a shifting of threshold [45]. A recent study by Samani et al. showed a decrease in the after-effect of using tDCS 20 min apart and on the following day [46]. In our study, we took precautions with the non-linear stimulus-response function, which is explained by the BCM theory as opposed to Hebbian synaptic plasticity, when we repeated the two adjacent stimulations, which may shift the resting threshold of another stimulation [30, 37, 38]. We shortened the pause duration between both stimulations as concisely as possible to avoid a sliding threshold of post-synaptic neuronal activity. To make certain that stimulation is concurrent with rehabilitation training in a non-homeostatic plasticity way.

We applied a single-time stimulation with the two techniques approach, which brought about a significant improvement in hand and arm functions in comparison with sham and lasted for at least 1 week. As mentioned before, we believe that the inhibitory LTD-like plasticity of glutamatergic synapses from Hebbian synaptic plasticity would play a crucial role in expanding the training effect. The long-term effect of NIBS would be induced by neuronal plasticity caused by inhibitory long-term depression (LTD) [18, 19] on the contralesional side, to regain balance and enhance excitability during skilled motor learning [20, 21] on the ipsilesional side. Once the patients started to benefit from the training effects of the skilled tasks, the memory remained at least for 1 week. We believe that this technique is promising and effective enough to enhance neural plasticity which lasts all through a week. It would play a crucial role in expanding the effect of stimulation from conventional rehabilitation training.

The results of this study should pave an alternative way for using NIBS as a method with greater efficiency, less resource usage, time, and budget-saving for patients with subacute stroke by reducing the number of stimulation sessions. In combination with conventional rehabilitation training, it should result in better neuronal plasticity in standard stroke rehabilitation.

Side effects detected in this study included headaches, neck pain, redness, and a burning sensation at the site of the electrode contact, which were all temporary. These were mild symptoms and recovered within 1 day after stimulation. No severe side effects, such as seizures, were seen in this study. The combined use of low-frequency rTMS and cathodal tDCS with patients with stroke was found in this study to be beneficial and safe [14–16, 23, 27, 31].

However, there were several limitations, in particular the sample size being small, which could possibly provide beta errors to the outcome measurement. No other objective outcomes were assessed, i.e., diagnostic TMS, EEG recording, or fMRI to evaluate the after-effects of the stimulation, owing to the variability of interindividual factors [47, 48], and the age effect [49, 50]. Apart from that, some studies even show the opposite direction of cortical excitability after stimulation [51, 52]. All of these constituents may interfere with non-homeostatic plasticity during training. No assessments for carry-over effects were performed for a period longer than 1 week to clarify how long the effects would persist. Even though we tried to have a double-blinded study, we did not collect a blinding success as a patient could not guess which was the active and which was a sham.

## Conclusions

One session of combined low-frequency rTMS with cathodal tDCS in the non-lesional primary motor cortex during the standard PT/OT program may enhance the function of the hands and arms in patients with subacute stroke. This can be reflected in the FMA-UE and WMFT scores immediately and up to 1 week after stimulation.

## Supplementary Information

**Additional file 1. **Statistical Analysis by SPSS version 25.0. **Supplementary 1.** Characteristics of Participating Patients at Baseline. **Supplementary 2.** Distribution of the Normalization Test. **Supplementary 3.** The 2-way mixed ANOVA of FMA-UE. **Supplementary 4.** Profile Plots Effects of Combined NIBS/Sham on FMA-UE. **Supplementary 5.** The 2-way mixed ANOVA of WMFT-FAS. **Supplementary 6.** Profile Plots Effects of Combined NIBS/sham on WMFT-FAS. **Supplementary 7.** The 2-way mixed ANOVA of WMFT-TIME. **Supplementary 8.** Profile Plots Effects of Combined NIBS/sham on WMFT-TIME.

**Additional file 2.** CONSORT Flow Diagram.

**Additional file 3.** CONSORT 2010 checklist.

**Additional file 4.** Registration and Trial protocol.

**Additional file 5.** Fugl-Meyer Assessment (FMA).

**Additional file 6.** Wolf Motor Function Test (WMFT).

**Additional file 7. **Subgroup analysis with adjusted baseline. **Supplementary 1.** Baseline-difference values for timepoints 1 (T1) and 2 (T2). **Supplementary 2.** The 2-way mixed ANOVA of FMA-UE with adjusted baseline. **Supplementary 3.** Post-hoc analysis using Bonferroni adjustment.

## Data Availability

All the data summarized and analyzed during this study are included in this published article; the original data from this study are available from the corresponding author upon reasonable request. See attachments for Additional files 1, 2, 3, 4, 5, 6 and 7.
