# Supplementary material for: Effects of combining two techniques of non-invasive brain stimulation in subacute stroke patients: a pilot study
Source: BMC Neurol. 2022 Mar 17;22:98. doi: 10.1186/s12883-022-02607-3 (PMC8928603; doi:10.1186/s12883-022-02607-3)
Supplement: Supplementary file 1 — Additional file 1. Statistical Analysis by SPSS version 25.0. Supplementary 1. Characteristics of Participating Patients at Baseline. Supplementary 2. Distribution of the Normalization Test. Supplementary 3. The 2-way mixed ANOVA of FMA-UE. Supplementary 4. Profile Plots Effects of Combined NIBS/Sham on FMA-UE. Supplementary 5. The 2-way mixed ANOVA of WMFT-FAS. Supplementary 6. Profile Plots Effects of Combined NIBS/sham on WMFT-FAS. Supplementary 7. The 2-way mixed ANOVA of WMFT-TIME. Supplementary 8. Profile Plots Effects of Combined NIBS/sham on WMFT-TIME. [file 12883_2022_2607_MOESM1_ESM.pdf]

Additional file 1_Statistical Analysis by SPSS version 25.0

Supplementary 1. Characteristics of Participating Patients at Baseline

Group Statistics

	group	N	Mean	Std. Deviation	Std. Error Mean
**Age**	1.00	5	58.8000	15.92796	7.12320
	2.00	5	59.2000	17.48428	7.81921
**Duration**	1.00	5	35.2000	21.67256	9.69226
	2.00	5	47.4000	25.03597	11.19643
**MRC-UE**	1.00	5	2.8000	1.09545	.48990
	2.00	5	2.2000	2.04939	.91652
**MRC-LE**	1.00	5	1.2000	1.30384	.58310
	2.00	5	2.0000	1.87083	.83666
**BS-ARM**	1.00	5	3.6000	.89443	.40000
	2.00	5	3.2000	1.09545	.48990
**BS-HAND**	1.00	5	2.8000	1.30384	.58310
	2.00	5	3.0000	1.00000	.44721
**FMA-UE**	1.00	5	23.4000	13.46477	6.02163
	2.00	5	21.4000	12.68069	5.67098
**WMFT-FAS**	1.00	5	28.4000	11.71751	5.24023
	2.00	5	28.2000	15.00667	6.71118
**WMFT-TIME**	1.00	5	87.6980	20.70791	9.26086
	2.00	5	89.5140	29.75848	13.30839

Group 1 = active, Group 2 = sham

Comment

Use descriptive statistics; mean and standard deviation for age, duration, MRC-UE, MRC-LE, BS-ARM, BS-HAND, FMA-UE, and WMFT.

Independent Samples Test

	Levene's Test for Equality of Variances		t-test for Equality of Means						
	F	Sig.	t	df	Sig. (2-tailed)	Mean Difference	Std. Error Difference	95% Confidence Interval of the Difference Lower	Upper

**Age**	Equal variances assumed	.439	.526	-.038	8	.971	-.40000	10.57733	-24.79138	23.99138
	Equal variances not assumed			-.038	7.931	.971	-.40000	10.57733	-24.82811	24.02811
**Duration**	Equal variances assumed	.042	.843	-.824	8	.434	-12.20000	14.80878	-46.34911	21.94911
	Equal variances not assumed			-.824	7.839	.434	-12.20000	14.80878	-46.47151	22.07151
**MRC-UE**	Equal variances assumed	8.511	.019	.577	8	.580	.60000	1.03923	-1.79647	2.99647
	Equal variances not assumed			.577	6.113	.584	.60000	1.03923	-1.93153	3.13153
**MRC-LE**	Equal variances assumed	2.420	.158	-.784	8	.455	-.80000	1.01980	-3.15167	1.55167
	Equal variances not assumed			-.784	7.144	.458	-.80000	1.01980	-3.20164	1.60164
**BS-ARM**	Equal variances assumed	1.440	.264	.632	8	.545	.40000	.63246	-1.05845	1.85845
	Equal variances not assumed			.632	7.692	.545	.40000	.63246	-1.06866	1.86866
**BS-HAND**	Equal variances assumed	.171	.690	-.272	8	.792	-.20000	.73485	-1.89456	1.49456
	Equal variances not assumed			-.272	7.496	.793	-.20000	.73485	-1.91459	1.51459
**FMA-UE**	Equal variances assumed	.040	.847	.242	8	.815	2.00000	8.27164	-17.07443	21.07443
	Equal variances not assumed			.242	7.971	.815	2.00000	8.27164	-17.08636	21.08636

**WMFT-FAS**	Equal variances assumed	.564	.474	.023	8	.982	.20000	8.51469	-19.43492	19.83492
	Equal variances not assumed			.023	7.556	.982	.20000	8.51469	-19.63770	20.03770
**WMFT-TIME**	Equal variances assumed	1.237	.298	-.112	8	.914	-1.81600	16.21348	-39.20435	35.57235
	Equal variances not assumed			-.112	7.138	.914	-1.81600	16.21348	-40.00500	36.37300

Comment

Test for differences in age, duration, MRC-UE, MRC-LE, BS-ARM, BS-HAND, FMA-UE, and WMFT via independent t-tests.

Crosstab

			Gender		
			1.00	2.00	Total
group	1.00	Count	1	4	5
		% within gender	50.0%	50.0%	50.0%
	2.00	Count	1	4	5
		% within gender	50.0%	50.0%	50.0%
Total		Count	2	8	10
		% within gender	100.0%	100.0%	100.0%

Chi-Square Tests

	Value	df	Asymptotic Significance (2-sided)	Exact Sig. (2-sided)	Exact Sig. (1-sided)
Pearson Chi-Square	.000[a]	1	1.000		
Continuity Correction[b]	.000	1	1.000		
Likelihood Ratio	.000	1	1.000		
Fisher's Exact Test				1.000	.778
Linear-by-Linear Association	.000	1	1.000		
N of Valid Cases	10				

a. 4 cells (100.0%) have expected count less than 5. The minimum expected count is 1.00.
b. Computed only for a 2x2 table

Crosstab

			Type of stroke		
			1.00	2.00	Total
group	1.00	Count	4	1	5
		% within type	50.0%	50.0%	50.0%
	2.00	Count	4	1	5
		% within type	50.0%	50.0%	50.0%
Total		Count	8	2	10
		% within type	100.0%	100.0%	100.0%

Chi-Square Tests

	Value	df	Asymptotic Significance (2-sided)	Exact Sig. (2-sided)	Exact Sig. (1-sided)
Pearson Chi-Square	.000[a]	1	1.000		
Continuity Correction[b]	.000	1	1.000		
Likelihood Ratio	.000	1	1.000		
Fisher's Exact Test				1.000	.778
Linear-by-Linear Association	.000	1	1.000		
N of Valid Cases	10				

a. 4 cells (100.0%) have expected count less than 5. The minimum expected count is 1.00.

b. Computed only for a 2x2 table

Crosstab

			Side of brain lesion		
			1.00	2.00	Total
group	1.00	Count	3	2	5
		% within side	50.0%	50.0%	50.0%
	2.00	Count	3	2	5
		% within side	50.0%	50.0%	50.0%
Total		Count	6	4	10
		% within side	100.0%	100.0%	100.0%

Chi-Square Tests

	Value	df	Asymptotic Significance (2-sided)	Exact Sig. (2-sided)	Exact Sig. (1-sided)

Pearson Chi-Square	.000[a]	1	1.000		
Continuity Correction[b]	.000	1	1.000		
Likelihood Ratio	.000	1	1.000		
Fisher's Exact Test				1.000	.738
Linear-by-Linear Association	.000	1	1.000		
N of Valid Cases	10				
a. 4 cells (100.0%) have expected count less than 5. The minimum expected count is 2.00.					
b. Computed only for a 2x2 table					

Crosstab

			Education				Total
			1.00	2.00	3.00	4.00	
group	1.00	Count	2	2	0	1	5
		% within education	50.0%	100.0%	0.0%	33.3%	50.0%
	2.00	Count	2	0	1	2	5
		% within education	50.0%	0.0%	100.0%	66.7%	50.0%
Total		Count	4	2	1	3	10
		% within education	100.0%	100.0%	100.0%	100.0%	100.0%

Chi-Square Tests

	Value	df	Asymptotic Significance (2-sided)
Pearson Chi-Square	3.333[a]	3	.343
Likelihood Ratio	4.499	3	.212
Linear-by-Linear Association	.503	1	.478
N of Valid Cases	10		
a. 8 cells (100.0%) have expected count less than 5. The minimum expected count is .50.			

Group 1 = active, Group 2 = sham

Comment

Use frequency to describe and test for differences in gender, type of stroke, side of brain lesion, and education via Chi-square and Fisher's Exact tests.

Supplementary 2. Distribution of the Normalization Test

Tests of Normality							
	Group	Kolmogorov-Smirnov[a]			Shapiro-Wilk		
		Statistic	df	Sig.	Statistic	df	Sig.
**FMA-UE before**	1	.334	5	.072	.813	5	.103
	2	.255	5	.200*	.828	5	.134
**FMA-UE immediately**	1	.323	5	.096	.812	5	.101
	2	.253	5	.200*	.822	5	.120
**FMA-UE 1 week**	1	.299	5	.165	.806	5	.090
	2	.259	5	.200*	.790	5	.067

Tests of Normality							
	Group	Kolmogorov-Smirnov[a]			Shapiro-Wilk		
		Statistic	df	Sig.	Statistic	df	Sig.
**WMFT-FAS before**	1	.336	5	.067	.795	5	.074
	2	.228	5	.200*	.883	5	.325
**WMFT-FAS immediately**	1	.275	5	.200*	.865	5	.246
	2	.232	5	.200*	.887	5	.342
**WMFT-FAS 1 week**	1	.268	5	.200*	.882	5	.318
	2	.249	5	.200*	.874	5	.283

Tests of Normality							
	Group	Kolmogorov-Smirnov[a]			Shapiro-Wilk		
		Statistic	df	Sig.	Statistic	df	Sig.
**WMFT-TIME before**	1	.318	5	.108	.804	5	.087
	2	.247	5	.200*	.819	5	.114
**WMFT-TIME immediately**	1	.248	5	.200*	.876	5	.291
	2	.256	5	.200*	.794	5	.073
**WMFT-TIME 1 week**	1	.242	5	.200*	.869	5	.263
	2	.256	5	.200*	.795	5	.073

Group 1 = active, Group 2 = sham

Comment

Data distribution testing, using the Shapiro-Wilk test, showed that the averages of FMA-UE, WMFT-FAS, and WMFT-TIME at three-time assessments were in a normal distribution.

Supplementary 3. The 2-way mixed ANOVA of FMA-UE

Mauchly's Test of Sphericity[a]							
Measure: **FMA-UE**							
					Epsilon[b]		
Within Subjects Effect	Mauchly's W	Approx. Chi-Square	df	Sig.	Greenhouse-Geisser	Huynh-Feldt	Lower-bound
factor1	.745	2.064	2	.356	.797	1.000	.500

factor 1 = ***FMA-UE***

Comment

Mauchly's Test of Sphericity indicated that the assumption of sphericity had not been violated, $\chi2(2) = 2.06$, $p = 0.36$.

Tests of Within-Subjects Effects						
Measure: **FMA-UE**						
Source		Type III Sum of Squares	df	Mean Square	F	Sig.
factor1	Sphericity Assumed	176.067	2	88.033	27.439	.000
	Greenhouse-Geisser	176.067	1.593	110.514	27.439	.000
	Huynh-Feldt	176.067	2.000	88.033	27.439	.000
	Lower-bound	176.067	1.000	176.067	27.439	.001
factor1 * Group	Sphericity Assumed	85.267	2	42.633	13.288	.000
	Greenhouse-Geisser	85.267	1.593	53.521	13.288	.001
	Huynh-Feldt	85.267	2.000	42.633	13.288	.000
	Lower-bound	85.267	1.000	85.267	13.288	.007
Error(factor1)	Sphericity Assumed	51.333	16	3.208		
	Greenhouse-Geisser	51.333	12.745	4.028		
	Huynh-Feldt	51.333	16.000	3.208		
	Lower-bound	51.333	8.000	6.417		

factor 1 = ***FMA-UE***

Comment

The overall FMA-UE motor score was statistically significant across three-time points, $F(2,16) = 27.44$, $p < 0.001$, a significant interaction between time and group of

experiments, $F(2,16) = 13.29$, $p < 0.001$ implying that the change in scores over time differed between group assigned.

Pairwise Comparisons

Measure: **FMA-UE**

Group	(I) time	(J) time	Mean Difference (I-J)	Std. Error	Sig.[b]	95% Confidence Interval for Difference[b]	
						Lower Bound	Upper Bound
1	1	2	-1.600	.812	.253	-4.050	.850
		3	-9.400*	1.323	.000	-13.389	-5.411
	2	1	1.600	.812	.253	-.850	4.050
		3	-7.800*	1.200	.001	-11.419	-4.181
	3	1	9.400*	1.323	.000	5.411	13.389
		2	7.800*	1.200	.001	4.181	11.419
2	1	2	-.600	.812	1.000	-3.050	1.850
		3	-1.800	1.323	.632	-5.789	2.189
	2	1	.600	.812	1.000	-1.850	3.050
		3	-1.200	1.200	1.000	-4.819	2.419
	3	1	1.800	1.323	.632	-2.189	5.789
		2	1.200	1.200	1.000	-2.419	4.819

Group 1 = active, Group 2 = sham, time 1 = at baseline, time 2 = immediately after stimulation, time 3 = 1 week after stimulation

Comment

Pairwise comparisons using Bonferroni's adjustment, only FM-UE of the active group had a significantly higher motor score at 1 week after stimulation, $p < 0.001$, as compared with the baseline and immediately after stimulation, $p = 0.001$.

Supplementary 4. Profile Plots Effects of Combined NIBS/Sham on FMA-UE

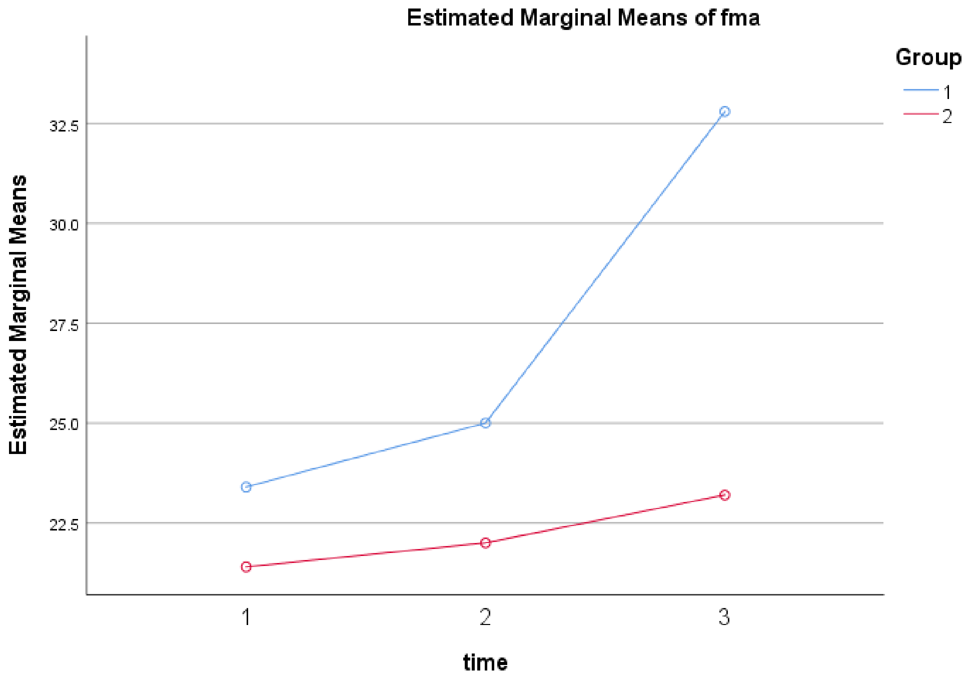

Group 1 = active, Group 2 = sham, 1 = at baseline, 2 = immediately after stimulation, 3 = 1 week after stimulation

Supplementary 5. The 2-way mixed ANOVA of WMFT-FAS

Mauchly's Test of Sphericity[a]							
Measure: **WMFT-FAS**							
					Epsilon[b]		
Within Subjects Effect	Mauchly's W	Approx. Chi-Square	df	Sig.	Greenhouse-Geisser	Huynh-Feldt	Lower-bound
factor1	.475	5.206	2	.074	.656	.831	.500

factor 1 = **WMFT-FAS**

Comment

Mauchly's Test of Sphericity indicated that the assumption of sphericity had not been violated, χ2(2) = 5.21, p = 0.07.

Tests of Within-Subjects Effects						
Measure: **WMFT-FAS**						
Source		Type III Sum of Squares	df	Mean Square	F	Sig.
time	Sphericity Assumed	130.467	2	65.233	13.879	.000
	Greenhouse-Geisser	130.467	1.312	99.459	13.879	.002
	Huynh-Feldt	130.467	1.662	78.488	13.879	.001
	Lower-bound	130.467	1.000	130.467	13.879	.006
factor1 * Group	Sphericity Assumed	31.667	2	15.833	3.369	.060
	Greenhouse-Geisser	31.667	1.312	24.141	3.369	.088
	Huynh-Feldt	31.667	1.662	19.050	3.369	.072
	Lower-bound	31.667	1.000	31.667	3.369	.104
Error(factor1)	Sphericity Assumed	75.200	16	4.700		
	Greenhouse-Geisser	75.200	10.494	7.166		
	Huynh-Feldt	75.200	13.298	5.655		
	Lower-bound	75.200	8.000	9.400		

factor 1 = **WMFT-FAS**

Comment

The 2-way mixed ANOVA was performed. The WMFT-FAS scores were statistically significant in time, $F(2,16) = 13.88$, $p < 0.001$. However, the time and group interactions were not statistically significant, even though there was an increase trend towards the experimental group, $F(2,16) = 3.37$, $p = 0.06$.

Supplementary 6. Profile Plots Effects of Combined NIBS/sham on WMFT-FAS

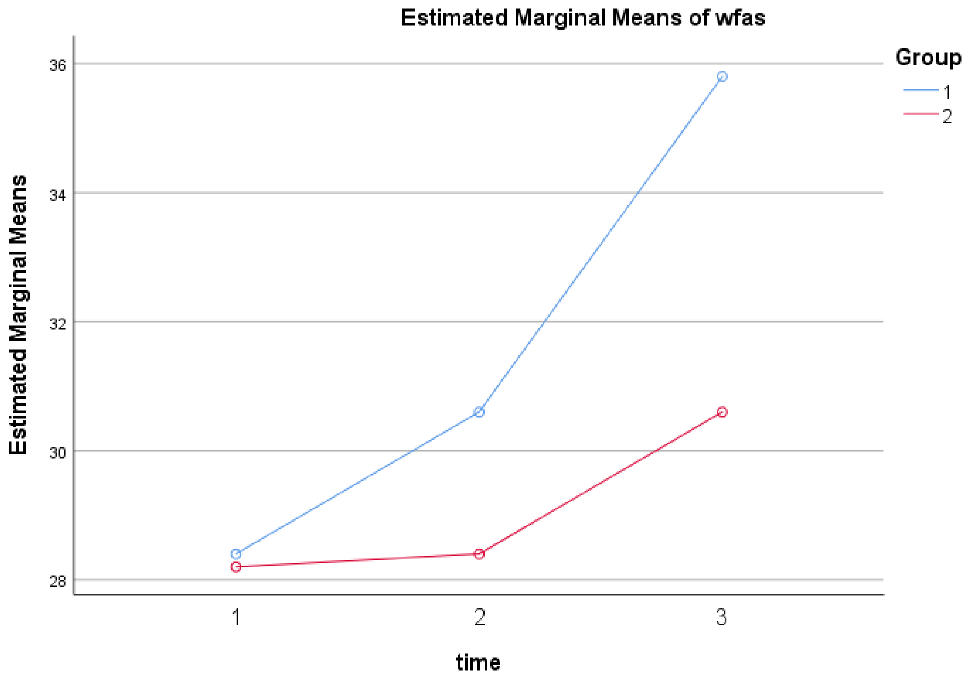

Group 1 = active, Group 2 = sham, 1 = at baseline, 2 = immediately after stimulation, 3 = 1 week after stimulation

Supplementary 7. The 2-way mixed ANOVA of WMFT-TIME

**Mauchly's Test of Sphericity[a]**							
Measure: **WMFT-TIME**							
					Epsilon[b]		
Within Subjects Effect	Mauchly's W	Approx. Chi-Square	df	Sig.	Greenhouse-Geisser	Huynh-Feldt	Lower-bound
time	.200	11.275	2	.004	.555	.661	.500

factor 1 = **WMFT-TIME**

Comment

Mauchly's Test of Sphericity indicated that the assumption of sphericity is violated, χ2(2) = 11.27, p = 0.004. We would prefer to use the lower-bound adjustment by Greenhouse-Geisser due to estimated epsilon (ε) is less than 0.75.

**Tests of Within-Subjects Effects**						
Measure: **WMFT-TIME**						
Source		Type III Sum of Squares	df	Mean Square	F	Sig.
factor1	Sphericity Assumed	656.881	2	328.441	5.148	.019
	Greenhouse-Geisser	656.881	1.111	591.273	5.148	.047
	Huynh-Feldt	656.881	1.322	496.759	5.148	.038
	Lower-bound	656.881	1.000	656.881	5.148	.053
factor1 * Group	Sphericity Assumed	484.381	2	242.190	3.796	.045
	Greenhouse-Geisser	484.381	1.111	436.001	3.796	.081
	Huynh-Feldt	484.381	1.322	366.307	3.796	.070
	Lower-bound	484.381	1.000	484.381	3.796	.087
Error(factor1)	Sphericity Assumed	1020.868	16	63.804		
	Greenhouse-Geisser	1020.868	8.888	114.863		
	Huynh-Feldt	1020.868	10.579	96.502		
	Lower-bound	1020.868	8.000	127.609		

factor 1 = **WMFT-TIME**

Comment

The 2-way mixed ANOVA was performed. The WMFT-TIME scores were statistically significant in time, $F(1.11, 8.89) = 5.15$, $p = 0.047$. However, the time and group

interactions were not statistically significant, even though there was an increase trend towards the experimental group, $F(1.11, 8.89) = 3.80$, $p = 0.08$.

Supplementary 8. Profile Plots Effects of Combined NIBS/sham on WMFT-TIME

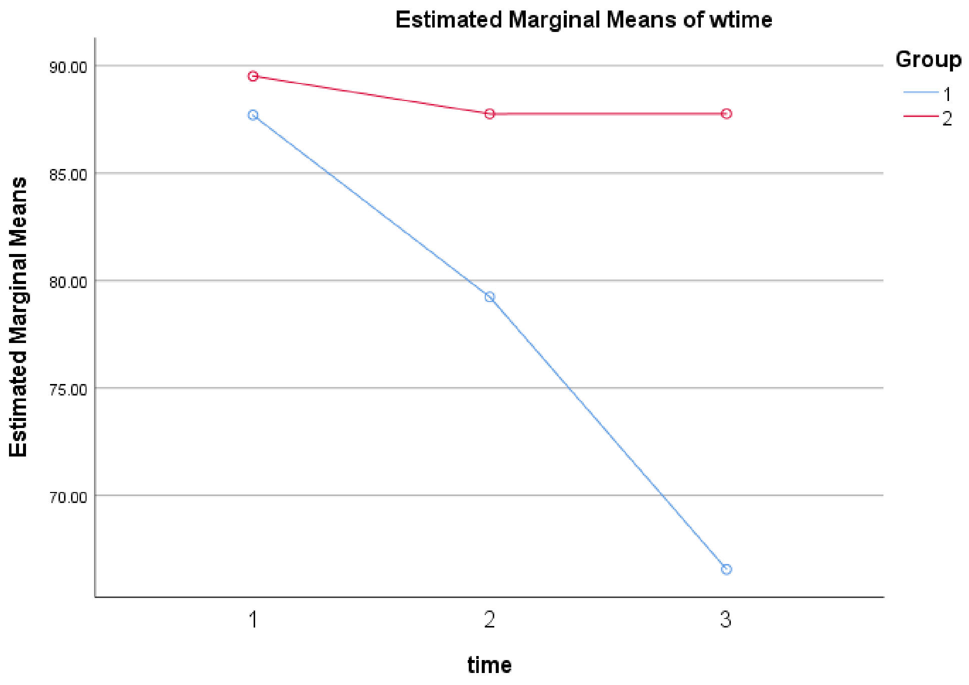

Group 1 = active, Group 2 = sham, 1 = at baseline, 2 = immediately after stimulation, 3 = 1 week after stimulation
