# Supplementary figures and images for: Effects of combining two techniques of non-invasive brain stimulation in subacute stroke patients: a pilot study
Source: BMC Neurol. 2022 Mar 17;22:98. doi: 10.1186/s12883-022-02607-3 (PMC8928603; doi:10.1186/s12883-022-02607-3)

**Additional file 2: CONSORT Flow Diagram of Patients Through the Study**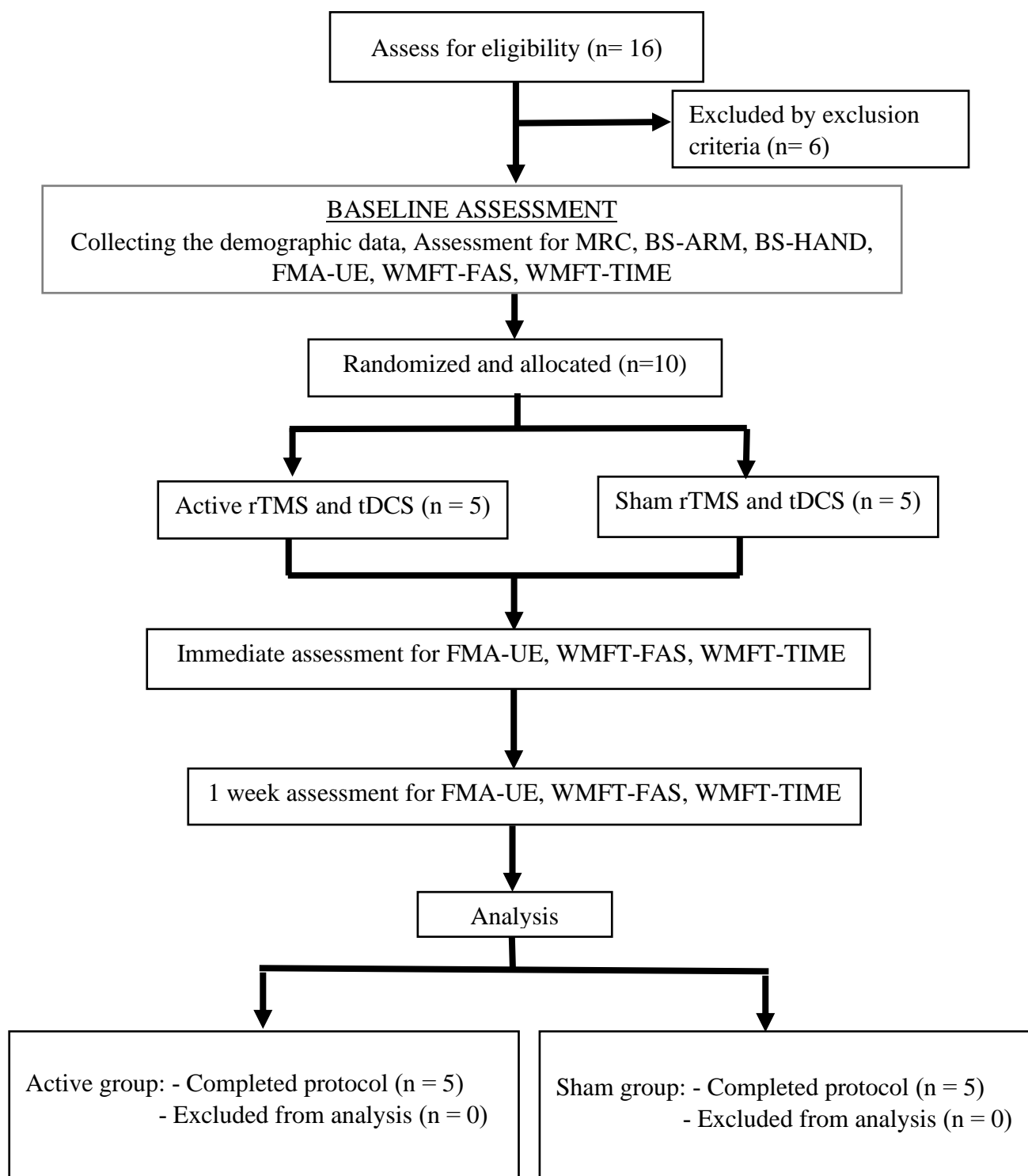

Supplement: Supplementary file 2 — Additional file 2. CONSORT Flow Diagram. [file 12883_2022_2607_MOESM2_ESM.pdf]
