# Supplementary material for: Effects of combining two techniques of non-invasive brain stimulation in subacute stroke patients: a pilot study
Source: BMC Neurol. 2022 Mar 17;22:98. doi: 10.1186/s12883-022-02607-3 (PMC8928603; doi:10.1186/s12883-022-02607-3)
Supplement: Supplementary file 4 — Additional file 4. Registration and Trial protocol. [file 12883_2022_2607_MOESM4_ESM.pdf]

Additional file 4_Registration and Trial protocol

Thai Clinical Trials Registry
www.thaiclinicaltrials.org

TCTR ID : TCTR20191027002

OTHER ID :

Overall Recruitment Status : Completed **(No Results)**

Retrospective registration
This protocol was registered after enrollment of the first participant.

Tracking Information

First Submitted Date :	18 October 2019
First Posted Date :	27 October 2019
Last Update Posted Date :	26 October 2019

Title

Public Title :	A combined effect of low-frequency repetitive transcranial magnetic stimulation (rTMS) and cathodal transcranial direct current stimulation (tDCS) over non-lesional primary motor cortex on upper limb motor recovery in subacute stroke
Acronym :	No Data
Scientific Title :	A combined effect of low-frequency repetitive transcranial magnetic stimulation (rTMS) and cathodal transcranial direct current stimulation (tDCS) over non-lesional primary motor cortex on upper limb motor recovery in subacute stroke
Sponsor ID/ IRB ID/ EC ID :	IRB/EC ID 409/2014
Registration Site :	Thai Clinical Trials Registry
URL :	https://www.thaiclinicaltrials.org/show/TCTR20191027002
Secondary ID :	-

Ethics Review

1. Board Approval :	Submitted, approved
Approval Number :	409/2014
Date of Approval :	No Data
Board Name :	Research Ethics Office, Faculty of Medicine, Chiang Mai University
Board Affiliation :	Faculty of Medicine, Chiang Mai University
Board Contact :	Business Phone : 0-5393-6643 Ext. No Data
	Business Email : Researchmed@cmu.ac.th
	Business Address : Faculty of Medicine 110 Si Phum Sub-district, Mueang Chiang Mai District, Chiang Mai 50200

Sponsor

Source(s) of Monetary or Material Supports :	Faculty of Medicine, Chiang Mai University
Study Primary Sponsor :	Faculty of Medicine, Chiang Mai University
Responsible Party :	Name/Official Title : Faculty of Medicine, Chiang Mai University
	Organization : Chiang Mai University
	Phone : 0-5393-6150 Ext. No Data
	Email : medinfo@cmu.ac.th
Study Secondary Sponsor :	N/A

Protocol Synopsis

Protocol Synopsis :

This document was created on 08 September 2021 at 13:56 (GMT+7).
