# Supplementary material for: Effects of combining two techniques of non-invasive brain stimulation in subacute stroke patients: a pilot study
Source: BMC Neurol. 2022 Mar 17;22:98. doi: 10.1186/s12883-022-02607-3 (PMC8928603; doi:10.1186/s12883-022-02607-3)
Supplement: Supplementary file 5 — Additional file 5. Fugl-Meyer Assessment (FMA). [file 12883_2022_2607_MOESM5_ESM.pdf]

Additional file 5_Fugl-Meyer Assessment (FMA)

Adapted from: Locomotor Experience Applied Post-Stroke (LEAPS) (NIH/NINDS/NCMRR R01 NS05056-01A1)
Sullivan et al for LEAPS Investigative Team

General Procedure and Rules

PROCEDURE	GENERAL RULES
**Description:** This assessment is a measure of upper extremity (UE) and lower extremity (LE) motor and sensory impairment.<br><br>**Equipment:** A chair, bedside table, reflex hammer, cotton ball, pencil, small piece of cardboard or paper, small can, tennis ball, stop watch, and blindfold.<br><br>**Administration:** The complete assessment usually requires 45 minutes.	Perform the assessment in a quiet area when the patient is maximally alert.<br><br>*Volitional movement assessment*: This includes flexor synergy, extensor synergy, movement combining synergies, movement out of synergy, wrist, hand, and coordination/speed. For all tests of volitional motion, these guidelines are to be followed:<br><br>1. Give clear and concise instructions. Mime as well as verbal instructions permissible.<br>2. Have patient perform the movement with non-affected extremity first. On affected side, check for available passive range of motion (PROM) prior to asking patient to perform the movement.<br>3. Repeat each movement 3x on the affected side and score best performance. If full score is attained on trials 1 or 2, do not have to repeat 3 times. Only test Coordination/speed, one time.<br>4. Do not assist patient, however verbal encouragement is permitted.<br>5. Test the wrist and hand function independently of the arm. **During the wrist tests (items 7a-e), support under the elbow may be provided to decrease demand at the shoulder; however, the patient should be activating the elbow flexors during the elbow at 90 degree tests and activating the elbow extensors during the elbow at 0 degree tests. In contrast, assistance can be provided to the arm at the elbow and just proximal to the wrist in order to position the arm during the hand tests (items 8a-g)**.

Fugl-Meyer Motor Assessment

Lower Extremity

Item	Procedure	Scoring
**I. Reflex activity**	• Patient is supine or sitting.<br>• Attempt to elicit the Achilles and patellar reflexes.<br>• Assess the unaffected side first.<br>• Test affected side.	• *Scoring* (Maximum possible score = 4):<br>• (0) - No reflex activity can be elicited;<br>• (2) - Reflex activity can be elicited. Items to be scored are Achilles and patellar reflexes.
**IIA. Flexor synergy**	• Patient is supine.<br>• Have patient perform movement with unaffected side first.<br>• On the affected side, check patient’s available PROM at each joint to be tested.<br>• Start with leg fully extended at hip, knee, and ankle. Instruct the patient to “bring your knee to your chest and	• *Scoring* (Maximum possible score = 6):<br>• (0) - Cannot be performed at all<br>• (1) – Partial motion<br>• (2) – Full motion

	pull up your toes" (therapist is observing for evidence of hip, knee, ankle flexion in order to assess the presence of all components of the flexor synergy). Therapist can cue the patient to move any missing component.<br>• Test 3x on the affected side and score best movement at each joint.	• Items to be scored are: Hip flexion, knee flexion, ankle dorsiflexion.
**IIB. <u>Extensor synergy</u>**	• Patient is sidelying.<br>• Have patient perform movement with unaffected side first.<br>• On the affected side, check patient's available PROM at each joint to be tested.<br>• Start in 90 degrees hip flexion, 90 degrees knee flexion and ankle dorsiflexion.<br>• Instruct the patient to "push your foot down and kick down and back". (Ankle plantarflexion, knee extension, hip adduction and hip extension.)<br>• Slight resistance should be applied in adduction which is gravity-assisted in this position to ensure patient is actively adducting.<br>• Test 3x on the affected side and score best movement at each joint.	• *Scoring* (Maximum possible score = 8):<br>• (0) – No motion<br>• (1) – Partial motion<br>• (2) – Full motion<br>• Items to be scored are: Hip extension, hip adduction, knee extension, ankle plantarflexion.
**III. <u>Movement combining synergies (in sitting)</u>**	<u>3a. Knee flexion beyond 90°:</u><br>• Patient is sitting, feet on floor, with knees free of chair. Knee to be tested is slightly extended beyond 90° knee flexion. Calf muscles should not be on stretch. To decrease friction, patient's shoes can be removed, but socks should remain on.<br>• Have patient perform movement with unaffected side first.<br>• Check patient's available PROM on the affected side for this motion.<br>• Patient is instructed to "pull your heel back and under the chair."<br>• Test 3x on the affected side and score best movement.	• *Scoring* (Maximum possible score = 2)*:*<br>• (0) – No active motion<br>• (1) – From slightly extended position, knee can be flexed but not beyond 90° or hip flexes while attempting to flex knee<br>• (2) – Knee flexion beyond 90°
	<u>3b. Ankle Dorsiflexion:</u><br>• Patient is sitting, feet on floor, with knees free of chair. Calf muscles should not be on stretch.<br>• Have patient perform movement with unaffected side first.<br>• On the affected side, check patient's available PROM at the ankle joint.<br>• Patient is instructed to "keeping your heel on the floor, lift your foot."<br>• Test 3x on the affected side and score best movement.	• *Scoring* (Maximum possible score = 2):<br>• (0) – No active motion<br>• (1) – Incomplete active flexion (heel must remain on floor with medial and lateral borders of the forefoot clearing the floor during dorsiflexion)<br>• (2) – Normal dorsiflexion (full within available ROM, heel remains on the floor)

**IV. Movement out of synergy (Standing, hip at 0 degrees)**	4a. Knee Flexion:<br>• Patient is standing, hip at 0 degrees (or full available ROM up to 0 degrees). On leg that is being tested, hip is at 0 degrees (or full available ROM up to 0 degrees), but the knee is flexed, and the patient's toes are touching the floor slightly behind. Evaluator can provide assistance to maintain balance and patient can rest hands on a table.<br>• Have patient perform movement with unaffected side first.<br>• Check patient's available PROM on the affected side for this motion.<br>• Patient is instructed to "keeping your hip back, kick your bottom with your heel."<br>• Test 3x on the affected side and score best movement.	• *Scoring* (Maximum possible score = 2):<br>• (0) – Knee cannot flex without hip flexion<br>• (1) – Knee flexion begins without hip flexion but does not reach to 90° or hip begins to flex in later phase of motion<br>• (2) – Knee flexion beyond 90° (Knee flexion beyond 90 degrees with hip maintained in extension)
**IV. Movement out of synergy (Standing, hip at 0 degrees)**	4b. Ankle Dorsiflexion:<br>• Patient is standing, hip at 0 degrees. If patient's calf muscle length is limiting active dorsiflexion in this starting position, then leg that is being tested can be positioned forward, so the hip is at approximately 5 degrees of flexion, and calf muscles are in lengthened position. Knee must stay fully extended. Evaluator can provide assistance to maintain balance and patient can rest hands on a table.<br>• Have patient perform movement with unaffected side first.<br>• On the affected side, check patient's available dorsiflexion PROM.<br>• Patient is instructed to "keeping your knee extended and your heel on the floor, lift your foot."<br>• Test 3x on the affected side and score best movement	• *Scoring* (Maximum possible score = 2):<br>• (0) – No active motion<br>• (1) – Partial motion (less than full available range with knee extended; heel must remain on floor with medial and lateral borders of the forefoot clearing the floor during dorsiflexion, or hip and/or knee flexes during motion while attempting dorsiflexion)<br>• (2) – Full motion (within available dorsiflexion range with knee extended and heel on the floor)
**V. Normal Reflexes (sitting)**	• This item is only included if the patient achieves a maximum score on all previous lower extremity items, otherwise score 0 .<br>• The examiner shall elicit patellar and Achilles phasic reflexes with a reflex hammer and knee flexors with quick stretch of the affected leg and note if the reflexes are hyperactive or not.	• *Scoring* (Maximum possible score = 2):<br>• (0) - At least 2 of the 3 phasic reflexes are markedly hyperactive<br>• (1) – One reflex is markedly hyperactive or at least 2 reflexes are lively<br>• (2) - No more than one reflex is lively and none are hyperactive

VI. **<u>Coordination/speed - Sitting: Heel to opposite knee repetitions in rapid succession</u>**	• Patient positioned in sitting with eyes open.<br>• Starting position is with heel to be tested resting on opposite ankle.<br>• Have patient perform movement with unaffected side first.<br>• Check available PROM on the affected side.<br>• Patient is instructed to "Bring your heel from your opposite ankle to your opposite knee, keeping your heel on your shin bone, move as fast as possible."<br>• Use a stopwatch to time how long it takes the patient to do 5 full (ankle to knee to ankle) repetitions.<br>• Use the full achieved active ROM on the unaffected limb as the comparison for the affected limb. If active ROM of affected limb is significantly less than that of unaffected limb, patient should be scored "0" for speed.<br>• Repeat the same movement with the affected leg. Record the time for both the unaffected and affected sides. Observe for evidence of tremor or dysmetria during the movement<br>• NOTE: This item attempts to discriminate between basal ganglia, thalamic, or cerebellar strokes in which tremor or dysmetria may result as a direct result of lesion to these areas. The majority of stroke cases are in the middle cerebral artery or basilar artery distributions where we expect to observe paralysis that affects movement speed but does not cause tremor or dysmetria. In cases of complete paralysis, observe for any indication of tremor or dysmetria that may be evident in face, voice, arms or legs. If there are no indicators of tremor or dysmetria, then score these items 2 and score speed 0.	• *Scoring Tremor* (Maximum possible score = 2):<br>◦ (0) - Marked tremor<br>◦ (1) – Slight tremor<br>◦ (2) – No tremor<br>• *Scoring Dysmetria* (Maximum possible score = 2):<br>◦ (0) - Pronounced or unsystematic dysmetria<br>◦ (1) – Slight or systematic dysmetria<br>◦ (2) – No dysmetria<br>• *Scoring Speed* (Maximum possible score = 2):<br>◦ (0) - Activity is more than 6 seconds longer than unaffected leg<br>◦ (1) – 2-5.9 seconds longer than unaffected leg<br>◦ (2) - less than 2 seconds difference

<table>
<tr><th colspan="3">Upper Extremity</th></tr>
<tr><th>Item</th><th>Instructions</th><th>Scoring</th></tr>
<tr><td>I. <u>Reflex activity</u></td><td>• Patient is sitting.
• Attempt to elicit the biceps and triceps reflexes.
• Test reflexes on unaffected side first.
• Test affected side.</td><td>• Scoring (Maximum possible score = 4):
  • (0) - No reflex activity can be elicited
  • (2) - Reflex activity can be elicited</td></tr>
<tr><td>II. <u>Flexor synergy</u></td><td>• Patient is sitting.
• Have patient perform movement with unaffected side first.
• On the affected side, check patient's available PROM at each joint to be tested.
• The starting position should be that of full extensor synergy. If the patient cannot actively achieve the starting position, the limb may be passively placed extended towards opposite knee in shoulder adduction/internal rotation, elbow extension, and forearm pronation.
• Instruct the patient to fully supinate his/her forearm, flex the elbow, and bring the hand to the ear of the affected side. The shoulder should be abducted at least 90 degrees.
• Test 3x on the affected side and score best movement at each joint</td><td>• Scoring (Maximum possible score = 12):
  • (0) - Cannot be performed at all
  • (1) - Performed partly
  • (2) - Performed faultlessly
• Items to be scored are: Elevation (scapular), shoulder retraction (scapular), shoulder abduction (at least 90 degrees) and external rotation, elbow flexion, and forearm supination.
  •</td></tr>
<tr><td>III. <u>Extensor synergy</u></td><td>• Patient is sitting.
• Have patient perform movement with unaffected side first.
• On the affected side, check patient's available PROM at each joint to be tested.
• The starting position should be that the limb is passively placed at patient's side in elbow flexion and supination. The examiner must ensure that the patient does not rotate and flex the trunk forward, thereby allowing gravity to assist with the movement. The pectoralis major and triceps brachii tendons may be palpated to assess active movement.
• Instruct the patient to adduct & internally rotate the shoulder, extend his arm towards the unaffected knee with the forearm pronated.
• Test 3x on the affected side and score best movement at each joint.</td><td>• Scoring (Maximum possible score = 6):
  • (0) - Cannot be performed at all
  • (1) - Performed partly
  • (2) - Performed faultlessly
• Items to be scored are: Shoulder adduction/internal rotation, elbow extension, and forearm pronation.</td></tr>
<tr><td>IV. <u>Movement combining synergies</u>
The patient is asked to perform</td><td>4a. Hand to lumbar spine:
• Patient is sitting with arm at side, shoulder at 0°, elbow at 0°.
• Have patient perform movement with unaffected side first.</td><td>• Scoring (Maximum possible score = 2):
  • (0) – No specific action is performed (or patient moves but does not reach</td></tr>
</table>

**three separate movements (4a, 4b, 4c).**	• Check patient's available PROM on the affected side for this motion.<br>• Patient is instructed to actively position the affected hand on the lumbar spine by asking them to "put your hand behind your back".<br>• Test 3x on the affected side and score best movement.	ASIS)<br>• (1) - Hand must pass anterior superior iliac spine (performed partly)<br>• (2) - Performed faultlessly (patient clears ASIS and can extend arm behind back towards sacrum; full elbow extension is not required to score a 2)
	4b. Shoulder flexion to 90°, elbow at 0°:<br>• Patient is sitting with hand resting on lap.<br>• Have patient perform movement with unaffected side first.<br>• On the affected side, check patient's available PROM for shoulder flexion to 90° and full elbow extension.<br>• Patient is instructed to flex the shoulder to 90°, keeping the elbow extended. The elbow must be fully extended throughout the shoulder flexor movement; the forearm can be in pronation or in a mid-position between pronation and supination.<br>• Test 3x on the affected side and score best movement.	• *Scoring* (Maximum possible score = 2):<br>• (0) – Arm is immediately abducted, or elbow flexes at start of motion<br>• (1) - Abduction or elbow flexion occurs in later phase of motion<br>• (2) - Performed faultlessly (patient can flex shoulder keeping elbow extended)
	4c. Pronation/supination of forearm, elbow at 90°, shoulder at 0°:<br>• Patient is sitting with arm at side, elbow flexed, and forearm in supination.<br>• Have patient perform movement with unaffected side first.<br>• On the affected side, check patient's available PROM for end range of pronation and supination.<br>• Patient is instructed to actively flex the elbow to 90° and pronate/supinate the forearm through the full available ROM.<br>• Test 3x on the affected side and score best movement.	• *Scoring* (Maximum possible score = 2):<br>• (0) – Correct position of shoulder held in adduction at side of body and elbow flexion, and/or pronation or supination cannot be performed.<br>• (1) – Active pronation or supination can be performed even within a limited range of motion, with elbow flexed at 90° and arm at side.<br>• (2) - Complete pronation and supination with with elbow flexed at 90° and arm at side.
**<u>V. Movement out of synergy</u>**<br><br>**The patient is asked to perform three separate movements (5a, 5b, 5c).**	5a. Shoulder abduction to 90°, elbow at 0°, and forearm pronated:<br>• Patient is sitting with arm and hand resting at side.<br>• Have patient perform movement with unaffected side first.<br>• Check patient's available PROM on the affected side for this motion.<br>• Patient is instructed to abduct the shoulder to 90°, in a pure abduction motion, with the elbow fully extended	• *Scoring* (Maximum possible score = 2):<br>• (0) – Initial elbow flexion occurs, or any deviation from pronated forearm occurs<br>• (1) - Motion can be performed partly, or, if during motion, elbow is

	and the forearm pronated.<br>• Test 3x on the affected side and score best movement.	flexed, or forearm cannot be kept in pronation;<br>• (2) - Performed faultlessly (patient can fully abduct shoulder, keeping forearm pronated with no elbow flexion)
	5b. Shoulder flexion from 90°-180°, elbow at 0°, and forearm in mid-position:<br>• Patient is sitting with elbow extended, hand resting on knee.<br>• Have patient perform movement with unaffected side first.<br>• Check patient's available PROM on the affected side for this motion.<br>• Patient is instructed to flex the shoulder above 90°, with the elbow fully extended and the forearm in the mid-position between pronation and supination.<br>• Test 3x on the affected side and score best movement.	• *Scoring* (Maximum possible score = 2):<br>• (0) – Initial flexion of elbow or shoulder abduction occurs (arm is immediately abducted, or elbow flexes at start of motion)<br>• (1) – Elbow flexion or shoulder abduction occurs during shoulder flexion (in later phases of motion)<br>• (2) - Performed faultlessly (patient can flex shoulder above, with forearm in mid-position and no elbow flexion)
	5c. Pronation/supination of forearm, elbow at 0°, and shoulder at 30°-90° of flexion:<br>• Patient is sitting with elbow extended, shoulder between 30°-90° of flexion.<br>• Have patient perform movement with unaffected side first.<br>• Check patient's available PROM on the affected side for this motion.<br>• Patient is instructed to pronate and supinate the forearm as the shoulder remains flexed between 30-90° and the elbow is fully extended.<br>• Test 3x on the affected side and score best movement.	• *Scoring* (Maximum possible score = 2):<br>• (0) – Supination and pronation cannot be performed at all, or elbow and shoulder positions cannot be attained<br>• (1) – Elbow and shoulder properly positioned and supination performed in a limited range<br>• (2) - Performed faultlessly (complete pronation and supination with correct positions at elbow and shoulder)
**VI. <u>Normal Reflexes</u> (sitting)**	• This item is only included if the patient achieves a maximum score on all previous upper extremity items, otherwise score 0.<br>• The examiner shall elicit biceps and triceps phasic reflexes with a reflex hammer and finger flexors with quick stretch and note if the reflexes are hyperactive or not.	• *Scoring* (Maximum possible score = 2):<br>• (0) - At least 2 of the 3 phasic reflexes are markedly hyperactive<br>• (1) – One reflex is markedly hyperactive or at least 2 reflexes are lively<br>• (2) - No more than one

		reflex is lively, and none are hyperactive
**<u>VII. Wrist</u>**<br><br>During the wrist tests, support under the elbow to may be provided to decrease demand at the shoulder; however, the patient should be activating the elbow flexors during the elbow at 90 degree tests and activating the elbow extensors during the elbow at 0 degree tests. The patient is asked to perform five separate movements (7a, 7b, 7c, 7d, 7e).	7a. Stability, elbow at 90°, and shoulder at 0°:<br>• Patient is sitting with arm and hand resting at side.<br>• Have patient perform movement with unaffected side first.<br>• Check patient's available PROM on the affected side for this motion.<br>• Patient is instructed to dorsiflex (extend) the wrist to the full range of 15° (or full available range) with the elbow at 90° flexion and the shoulder at 0°. If full range of dorsiflexion is attained, slight resistance is given.<br>• Test 3x on the affected side and score best movement.	• *Scoring* (Maximum possible score = 2):<br>• (0) - Patient cannot dorsiflex wrist to required 15°<br>• (1) – Dorsiflexion is accomplished, but no resistance is taken<br>• (2) - Position can be maintained with some (slight) resistance
	7b. Flexion/extension, elbow at 90°, and shoulder at 0°:<br>• Patient is sitting with arm and hand resting at side.<br>• Have patient perform movement with unaffected side first.<br>• Patient is instructed to perform repeated smooth alternating movements from 15 degrees of flexion (wrist extension) to 15 degrees of extension.<br>• Test 3x on the affected side and score best movement	• *Scoring* (Maximum possible score = 2):<br>• (0) - Volitional movement does not occur<br>• (1) – Patient cannot actively move through the wrist joint throughout the total range of motion<br>• (2) – Faultless, smooth movement (repetitive through full available ROM)
	7c. Stability, elbow at 0°, and shoulder at 30° flexion:<br>• Patient is sitting with elbow extended, hand resting on knee and forearm pronated.<br>• Have patient perform movement with unaffected side first.<br>• Check patient's available PROM on the affected side for this motion.<br>• Patient is instructed to dorsiflex (extend) the wrist to the full range of 15° (or full available range) with the elbow fully extended and the shoulder at 30° flexion. If full range of dorsiflexion is attained, slight resistance is given.<br>• Test 3x on the affected side and score best movement.	• *Scoring* (Maximum possible score = 2):<br>• (0) - Patient cannot dorsiflex wrist to required 15°<br>• (1) – Dorsiflexion is accomplished, but no resistance is taken<br>• (2) - Position can be maintained with some (slight) resistance
	7d. Flexion/extension, elbow at 0°, and shoulder at 30° flexion:<br>• Patient is sitting with elbow extended, hand resting on knee and forearm pronated.<br>• Have patient perform movement with unaffected side first.<br>• Patient is instructed to perform repeated smooth alternating movements from maximum dorsiflexion to maximum volar flexion with the fingers somewhat flexed to the full range of 15° (or full available range)	• *Scoring* (Maximum possible score = 2):<br>• (0) - Volitional movement does not occur<br>• (1) – Patient cannot actively move throughout the total range of motion;<br>• (2) – Faultlessly, smooth movement (repetitive through full ROM)

	with the elbow fully extended and the shoulder at 30° flex.<br>• Test 3x on the affected side and score best movement.	
	7e. Circumduction:<br>• Patient is sitting with arm at side elbow flexed to 90°, and forearm pronated.<br>• Have patient perform movement with unaffected side first.<br>• Check patient's available PROM on the affected side for this motion.<br>• Patient is instructed to circumduct the wrist with smooth alternating movements throughout the full range of circumduction.<br>• Test 3x on the affected side and score best movement.	• *Scoring* (Maximum possible score = 2):<br>• (0) – Cannot be performed (volitional movement does not occur)<br>• (1) – Jerky motion or incomplete circumduction<br>• (2) – Complete motion with smoothness (performs faultlessly, smooth, repetitive movement through full ROM)
**VIII. Hand**<br><br>During the hand tests, assistance can be provided to the arm at the elbow and just proximal to the wrist in order to position the arm for the grasp tasks.<br>The patient is asked to perform seven separate movements (8a, 8b, 8c, 8d, 8e, 8f, 8g). The object is not placed in the hand but presented to the patient so that it requires sufficient opening to grasp test object, closure on object, ability to hold against a slight tug.	8a. Finger mass flexion:<br>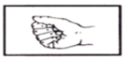<br>• Patient is sitting with arm on bedside table or lap.<br>• Have patient perform movement with unaffected side first.<br>• Check patient's available PROM on the affected side for this motion.<br>• Starting from the position of finger extension (this may be attained passively if necessary), instruct the patient to fully flex all fingers.<br>• Test 3x on the affected side and score best movement	• *Scoring* (Maximum possible score = 2):<br>• (0) – No flexion occurs<br>• (1) – Some flexion, but not full motion<br>• (2) – Completed active flexion (compared to unaffected hand)
	8b. Finger mass extension:<br>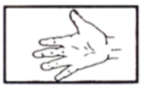<br>• Patient is sitting with arm on bedside table or lap.<br>• Have patient perform movement with unaffected side first.<br>• Check patient's available PROM on the affected side for this motion.<br>• Starting from the position of finger flexion (this may be attained passively if necessary), instruct the patient to fully extend all fingers.<br>• Test 3x on the affected side and score best movement.	• *Scoring* (Maximum possible score = 2):<br>• (0) – No extension occurs<br>• (1) – Patient can release an active mass flexion grasp<br>• (2) – Full active extension (compared to unaffected side)
	8c. Grasp I:<br>• Patient is sitting with arm on bedside table.<br>• Have patient perform movement with unaffected side first.<br>• Check patient's available PROM on the affected side for	• *Scoring* (Maximum possible score = 2):<br>• (0) – Required position cannot be attained<br>• (1) – Grasp is weak

	this motion.<br>• Instruct the patient to extend the metacarpophalangeal joints of digits II-V and flex the proximal & distal interphalangeal joints. Test this grip against resistance. You can tell the patient "pretend you are holding the handle of a briefcase."<br>• Test 3x on the affected side and score best movement.	• (2) – Grasp can be maintained against relatively great resistance
	8d. Grasp II:<br>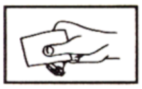 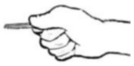<br>•<br>• Patient is sitting with arm on bedside table.<br>• Have patient perform movement with unaffected side first.<br>• Instruct the patient to abduct the thumb to grasp a piece of paper. Then ask the patient to perform pure thumb adduction with the scrap of paper interposed between the thumb and first digit (as in figure). Test this grip against resistance by asking the patient to hold as you attempt to pull the paper out with a slight tug.<br>• Test 3x on the affected side and score best movement.	• *Scoring* (Maximum possible score = 2):<br>• (0) – Function cannot be performed<br>• (1) – Scrap of paper interposed between the thumb and index finger can be kept in place, but not against a slight tug<br>• (2) – Paper is held firmly against a tug
	8e. Grasp III:<br>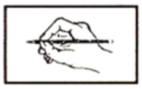 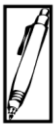<br>• Patient is sitting with arm on bedside table.<br>• Have patient perform movement with unaffected side first.<br>• Instruct the patient to grasp a pen or pencil by opposing the thumb and index finger pads around the pen. The tester may support the patient's arm but may not assist with the hand function required for the retrieval task. The pen may not be stabilized by the therapist or the patient's other hand. To minimize excessive movement, however, a pen with a 'pocket clip' that prevents rolling more than 180° may be used.<br>• Once the pencil is retrieved, instruct the patient to oppose the thumb pad against the pad of the index finger with a pencil interposed. Test this grip against resistance by asking the patient to hold as you attempt to pull the pencil out with a slight tug upwards.<br>• Test 3x on the affected side and score best movement.	• *Scoring* (Maximum possible score = 2):<br>• (0) – Function cannot be performed<br>• (1) – A pencil interposed between the thumb pad and the pad of the index finger can be kept in place, but not against a slight tug<br>• (2) – Pencil is held firmly against a tug
	8f. Grasp IV:<br>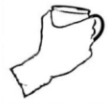<br>• Patient is sitting with arm on bedside table.	• *Scoring* (Maximum possible score = 2):<br>• (0) Function cannot be performed<br>• (1) – A can interposed between the thumb and

	• Have patient perform movement with unaffected side first.<br>• Instruct the patient to grasp a small can (placed upright on a table without stabilization) by opening the fingers and opposing the volar surfaces of the thumb and digits. The arm may be supported but the tester may not assist with hand function.<br>• Once the can is grasped, test this grip against resistance by asking the patient to hold as you attempt to pull the can out with a slight tug. Test 3x on the affected side and score best movement.	index finger can be kept in place, but not against a slight tug<br>• (2) – Can is held firmly against a tug<br>• NOTE: the hand must open and close on the can; it is not acceptable to have the patient grasp can by coming down from the top of the can.
	8g. Grasp V: 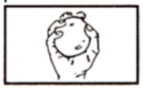 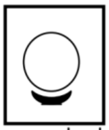<br>• Patient is sitting with arm on bedside table.<br>• Have patient perform movement with unaffected side first.<br>• Instruct the patient to perform a spherical grasp by grasping a tennis ball The tester may support the patient's arm but may not assist with the hand function required for the retrieval task. The ball may not be stabilized by the therapist or the patient's other hand. To minimize excessive movement, the ball can be placed on an object that reduces rolling. An inverted medium-sized bottle cap placed under the ball to prevent rolling is acceptable. Once the tennis ball is grasped, test this grip against resistance by asking the patient to hold as you attempt to pull the ball out with a slight tug.<br>• Test 3x on the affected side and score best movement.	• *Scoring (*Maximum possible score = 2):<br>• (0) Function cannot be performed<br>• (1) – A tennis ball can be kept in place with a spherical grasp, but not against a slight tug<br>• (2) – Tennis ball is held firmly against a tug
**IX. Coordination and speed - Sitting: Finger to nose (5 repetitions in rapid succession)**	• Patient positioned in sitting with eyes open.<br>• Starting position is with hand on lap.<br>• Have patient perform movement with unaffected side first.<br>• Check patient's available PROM on the affected side for this motion.<br>• Patient is instructed to "bring your finger from your knee to your nose, as fast as possible."<br>• Use a stopwatch to time how long it takes the patient to do 5 repetitions.<br>• Repeat the same movement with the affected arm. Record the time for both the unaffected and affected sides. Observe for evidence of tremor or dysmetria during the movement.<br>• NOTE: This item attempts to discriminate between basal ganglia, thalamic, or cerebellar strokes in which tremor or dysmetria may result as a direct result of lesion to these areas. The majority of stroke cases are	• *Scoring Tremor* (Maximum possible score = 2):<br>• (0) - Marked tremor<br>• (1) – Slight tremor<br>• (2) – No tremor<br>• *Scoring Dysmetria* (Maximum possible score = 2):<br>• (0)- Pronounced or unsystematic dysmetria<br>• (1) – Slight or systematic dysmetria<br>• (2) – No dysmetria<br>• *Scoring Speed* (Maximum possible score = 2):<br>• (0) – Activity is more than 6 seconds longer than unaffected hand<br>• (1) – (2-5.9) seconds longer

	in the middle cerebral artery or basilar artery where we expect to observe paralysis that affects movement speed but does not cause tremor or dysmetria. In cases of complete paralysis, observe for any indication of tremor or dysmetria that may be evident in face, voice, arms or legs. If there are no indicators of tremor or dysmetria, then score these items 2 and score speed 0. If active ROM of affected limb is significantly less than that of affected limb, patient should be scored "0" for speed.	than unaffected side<br>• (2) – less than 2 seconds difference

Adapted from: Locomotor Experience Applied Post-Stroke (LEAPS)
(NIH/NINDS/NCMRR R01 NS05056-01A1)
Sullivan et al for LEAPS Investigative Team
