# Supplementary material for: Effects of combining two techniques of non-invasive brain stimulation in subacute stroke patients: a pilot study
Source: BMC Neurol. 2022 Mar 17;22:98. doi: 10.1186/s12883-022-02607-3 (PMC8928603; doi:10.1186/s12883-022-02607-3)
Supplement: Supplementary file 6 — Additional file 6. Wolf Motor Function Test (WMFT). [file 12883_2022_2607_MOESM6_ESM.pdf]

Additional file 6_Wolf Motor Function Test (WMFT)

Adapted from © 2011 UAB CI Therapy Research Group

Task Instructions

I. ***Two Tasks (IA. & IB. – Tasks 1 & 2).*** **Functional ability of the shoulder of the involved upper extremity; tasks performed to the side of the patient (i.e., away from the midsagittal plane of the patient). Shoulder movement of abduction.**

IA. (1). Forearm to table (side)

SET UP	TASK	VERBAL INSTRUCTIONS
Starting Position:<br><br>• Chair Position (Side).<br>• Hips against back of chair.<br>• Hands in lap.<br>• Both feet on floor.<br>• Filming Position (Side).	Task Description:<br><br>Patient attempts to place forearm on the table (adjacent and parallel to front edge) by abduction at the shoulder. (Some shoulder flexion will probably also be necessary to get arm past the edge of table.)<br>"Forearm" is defined as the wrist and elbow. The palmar surface of the hand need not be flat. Timing ends when both the forearm and hand touch the table.<br><br>Timing Procedure:<br><br>Starts on word "Go" and ends when patient's forearm and hand both touch the table in the required position.<br><br>Measure:<br><br>The time elapsed from the starting point to the moment the forearm and hand touch the table in the required fashion.	Verbal Instructions:<br><br>• "Place your forearm on the table as quickly as you can. Do it just like this (examiner demonstrates). At the end of the movement, your forearm and hand should be touching the surface of the table. Do this as quickly as you can." (repeat instructions)<br>• "Do you have any questions?"<br>• "Ready, set, go."<br><br>Scoring:<br><br>FA scoring should take into account the extent to which the head and trunk are maintained in normal alignment and the speed, fluidity, and precision with which movements are performed.

IB (2). Forearm to box (side)

<table>
<tr><th>SETUP</th><th>TASK</th><th>VERBAL INSTRUCTIONS</th></tr>
<tr><td>Starting Position:

• Chair Position (Side).
• Hips against back of chair.
• Hand not being tested in lap.
• Shoulder of tested arm abducted with the forearm pronated and placed flat on table with radial edge adjacent to front edge of table; elbow at line 14-cm. from side edge of template. Palmar surface of hand need not to be flat. If final position of arm on previous task (IA) is not 14-cm. from side edge of table, move subject's arm into correct position before beginning this task.
• Place a box of appropriate height (See comment #16) in the template area located 13.5-cm. from the edge of the template and 13.5-cm. from the midline. Box should be stabilized by someone during the trial.
• Filming Position (Side).</td><td>Task Description:

Patient attempts to place forearm (from wrist to elbow) on the box by further abduction at the shoulder. (Again some shoulder flexion may be necessary to clear edge of box.) At the end, the forearm should be flat on the box with the hand drooping over side edge of box. The wrist must be beyond the line 2-cm. from the front edge of box and the elbow must be beyond the front edge of the box.

Timing Procedure:

Starts on word "Go" and ends when patient's forearm and elbow are flat on the box, wrist is beyond 2-cm. line and the hand is beyond the end of the box in a relaxed position.

Measure:

The time elapsed from starting point to the moment the forearm touches the top of the box in the required fashion with the hand drooping over the edge of the box.</td><td>Verbal Instructions:

• "Place your forearm on the box as quickly as you can. Do it just like this (demonstrate). At the end, your whole forearm should be flat and touching the surface of the box and your hand drooping over the edge of the box. Your wrist is beyond this line and your elbow must be completely on the surface of the box. Do this as quickly as you can." (repeat instructions)
• "Do you have any questions?"
• "Ready, set, go."

Scoring:

FA scoring should take into account the extent to which the head and trunk are maintained in normal alignment and the speed, fluidity, and precision with which movements are performed.</td></tr>
</table>

II. ***<u>Two Tasks (IIA. & IIB. – Tasks 3&4)</u>*. Functional ability of the elbow of the involved upper extremity; movements performed to the side of the patient (i.e., away from the midsagittal plane). Elbow movements of extension. (A small amount of external rotation at shoulder is a necessary component of these two tasks, but elbow extension is the primary component.)**

<u>IIA (3). Extend elbow (to the side)</u>

SET UP	TASK	VERBAL INSTRUCTIONS
<u>Starting Position:</u><br>• Chair Position (Side).<br>• Hips against back of chair.<br>• Table surface should be lightly dusted with baby/talcum powder.<br>• Hand not being tested in lap.<br>• Shoulder of tested arm abducted with forearm resting flat on table in a pronated position. Palmar surface of hand need not be flat on table.<br>• Forearm being tested adjacent to front edge of table; elbow at line 14-cm from side edge of template.<br>• Filming Position (Side).	<u>Task description:</u><br>Patient attempts to reach across the 40-cm. line on template by extending the elbow (to the side). Elbow can be lifted off the table during the task. This may be the only way shorter subjects can reach 40-cm. line. Shoulders should be kept level to prevent leaning with the trunk. Some external rotation at the shoulder is necessary to carry out this movement, but the examiner should prevent too much of this movement.<br><u>Timing Procedure:</u><br>Starts on the word "Go" and ends when the patient's thumb passes the line.<br><u>Measure</u>:<br>The time elapsed from the starting point to the time the thumb crosses the line.	<u>Verbal Instructions:</u><br>• "Slide your hand across the table by moving your hand away from your body and straighten your elbow to its fullest extent. Your thumb should cross this line (point to the 40-cm line). You can raise your elbow from the table if you like. Also, please keep your shoulders level and just move your arm, just like this (demonstrate). Do not lean over; keep your body as straight as possible. Do this as quickly as you can." (repeat instructions)<br>• Note: the patient should <u>slide</u> their hand across the table. Repeat the task if they lift their hand off of the table.<br>• "Do you have any questions?"<br>• "Ready, set, go."<br><u>Scoring:</u><br>• FA scoring should take into account: 1) the extent to which the head and trunk are maintained in normal alignment, 2) whether the elbow is extended, 3) whether the hand remains in contact with the table, and 4) the speed, fluidity, and precision with which movements are performed.<br>• The elbow can be lifted off the table. Also, some shoulder external rotation and abduction is necessary, but inadequate or excessive motions of this type should be noted.

IIB (4). Extend elbow (to the side) - with weight

SET UP	TASK	VERBAL INSTRUCTIONS
Starting Position:<br><br>• Chair Position (Side).<br>• Hips against back of chair.<br>• Hand not being tested in lap.<br>• Shoulder of tested arm abducted with forearm resting flat on table in pronated position exactly as in last task.<br>• Forearm of arm to be tested adjacent to front edge of table; elbow at line 14-cm. from side edge of template; palmar surface of hand need not be flat.<br>• 1 lb. weight placed at ulnar edge of wrist; distal end of the weight is aligned with ulnar styloid process (i.e., the weight is only touching the forearm).<br>• Filming Position (Side).	Task description:<br><br>Patient attempts to push the weight across 40-cm. line by extending the elbow and (to a lesser extent) externally rotating shoulder. Elbow should be kept on the table throughout the task (different from the previous task), and shoulders should be kept level to prevent leaning with the trunk. Again, the examiner needs to be aware of patient's trunk leaning and/or excessive external rotation at the shoulder to perform task (especially true for taller men). Note: the weight is to remain in contact with the forearm throughout the task. Repeat the task if the subject swats the weight.<br><br>Timing Procedure:<br><br>Starts on the word "Go" and ends when any part of the weight crosses line.<br><br>Measure:<br><br>The time elapsed from the starting point to the time the leading edge of the weight initially crosses the line.	Verbal Instructions:<br><br>• "Push the weight across the line (point to 40-cm line) by moving your hand away from your body while trying to keep your elbow on the table. Your forearm should remain in contact with the weight until the weight crosses the line. Also, again, please keep your shoulders level and just move your arm, just like this (demonstrate). Do not lean over; keep your body straight. Do this as quickly as you can." (repeat instructions)<br>• "Do you have any questions?"<br>• "Ready, set, go."<br><br>Scoring:<br><br>• FA scoring should take into account: 1) the extent to which the head and trunk are maintained in normal alignment, 2) whether the forearm remains in contact with the weight, and 3) the speed, fluidity, and precision with which movements are performed.<br>• Some shoulder abduction is necessary, but inadequate or excessive motions of this type should be noted.<br>• If the forearm doesn't remain in contact with the weight, a maximum score of 3 should be assigned.<br>• If accomplished with excessive compensatory trunk movement and/or very limited elbow extension, a maximum score of 2 should be assigned.

III. ***Three Tasks (IIIA., IIIB., & IIIC. – Tasks 5,6,&7).*** **Functional ability of the shoulder of the involved upper extremity; performed to the front of the patient.**

IIIA(5). Hand to table (front)

SET UP	TASK	VERBAL INSTRUCTIONS
Starting Position:<br><br>• Chair Position (Front).<br>• Both hands in lap.<br>• Hips against back of chair.<br>• Patient positioned so that leaning is not necessary to comfortably reach the table.<br>• Filming Position (Side).	Task Description:<br><br>Patient attempts to place hand being tested on the table. The heel of the hand must rest beyond the line 2 cm. from front edge of table. The palmar surface of the hand need not be flat. (The subject should place most of the hand in the circle.)<br><br>Timing Procedure:<br><br>Starts on the word "Go" and ends when the heel of the hand and fingers touch table beyond the taped 2-cm. line.<br><br>Measure:<br><br>The time elapsed from starting point to moment the heel of the hand and fingers touch table beyond the taped 2 cm. line.	Verbal Instructions:<br><br>• "Place your hand on the table so that the heel of your hand is beyond the 2-cm. line. Most of your hand should be placed in the circle (demonstrate). Your hand does not need to be flat. Do this as quickly as you can." (repeat instructions)<br>• "Do you have any questions?"<br>• "Ready, set, go."<br><br>Scoring:<br><br>• FA scoring should take into account the extent to which the head and trunk are maintained in normal alignment and the speed, fluidity, and precision with which movements are performed.<br>• **Note**: The final posture of the hand and fingers does not influence scoring as long as the heel of the hand is in contact with the table.

IIIB(6). Hand to box (front)

SET UP	TASK	VERBAL INSTRUCTIONS
Starting position:<br><br>• Chair Position (Front).<br>• Hips against back of chair.<br>• Hand not being tested in lap.<br>• Hand to be tested placed on table, heel of hand just beyond the line 2 cm. from front edge of table (i.e., just past line, in circle - as in final position on last task).<br>• Box centered on table; front edge aligned with 20-cm. line. Box should be stabilized by someone during the trial.<br>• Filming Position (Side).	Task Description:<br><br>Patient attempts to place hand on the box. The heel of the hand must be placed past the front edge of box. The palmar surface of the hand need not be flat.<br><br>Timing Procedure:<br><br>Starts on the word "Go" and ends when the heel of hand and fingers touch the box past the front edge of box.<br><br>Measure:<br><br>The time elapsed from starting point to moment the heel of hand and fingers touch box past the edge of the box.	Verbal Instructions:<br><br>• "Lift your hand from the table and place it on the box so that the heel of your hand goes past the edge of the box (demonstrate). Do this as quickly as you can." (repeat instructions)<br>• "Do you have any questions?"<br>• "Ready, set, go."<br><br>Scoring:<br><br>• FA scoring should take into account the extent to which the head and trunk are maintained in normal alignment and the speed, fluidity, and precision with which movements are performed.<br>• **Note:** The final posture of the hand and fingers does not interfere with scoring as long as the heel of the hand is in contact with the box.

IIIC (7). Weight to box.

SETUP	TASK	VERBAL INSTRUCTIONS
Starting Position:<br><br>• Chair Position (Front).<br>• Hips against back of chair.<br>• Hands not to be tested in lap.<br>• Heel of hand to be tested placed on table just beyond 2-cm. taped line.<br>• Cuff weight(s) in place around forearm to be tested; stacking of weights begun just distal to the elbow. The weights should be stacked while arm to be tested is resting on the table to avoid fatiguing the arm.<br>• Cuff weights with 1-lb. inserts are preferable.<br>• Box centered on table; front edge aligned with 20-cm. line. Box should be stabilized by someone during the trial.<br>• Not Filmed.	Task Description:<br><br>Patient attempts to place the (weighted) hand being tested on the box so that the heel of hand rests beyond the front edge of the box. Patient should not be permitted to lean in and use their body to help lift the weight; their entire back should remain in contact with the chair (scapular protraction of the moving UE is allowed). Tester may, place their finger behind the subject's back at the top of the chair to determine if the subject's back moves away from the chair. In stacking the weights near wrist, be sure to leave enough room for weights to clear the table.<br><br>Timing Procedure:<br><br>Not applicable.<br><br>Measure:<br><br>• Amount of weight patient is able to lift to the box while keeping their back against the chair (not timed as previous tasks).<br><br>• Weight amounts in the order attempted should be recorded so that the pre-treatment order can be duplicated post-treatment. The maximum amount of weight lifted should be circled.	Verbal Instructions:<br><br>• "Place your hand on the box so that the heel of your hand is beyond the front edge of the box (demonstrate). Keep your back against the chair. That is very important. Take your time. You do not need to hurry." (repeat instructions)<br>• "Do you have any questions?"<br>• "Ready, set, go."<br><br>Special Considerations:<br><br>• For the initial trial, the examiner should subjectively determine the appropriate starting weight by resisting the patient's attempt to hold the elbow extended, shoulder flexed to 90^0. The stronger the patient appears, the higher the initial weight should be. If patient is weak, task should begin with lower initial weight. Increases in the amount of 2 lbs. should continue until the patient's maximum or 20 lbs. is reached. When the patient has reached his/her apparent maximum, the next trial should be one lb. less. If that weight can be lifted, it is recorded as the maximum. A 2-minute rest period should be allowed after every three trials.<br>• Repeat the same sequence of weights for each subsequent testing session to keep the effects of fatigue consistent across assessments.<br>• Tester may place their finger behind the patient's back at the top of the chair to determine if the subject's back leaves the chair.

IV. ***<u>One Task (IVA. – Task 8).</u>*** **Functional ability of the elbow of the involved upper extremity; performed to the front of the patient.**

<u>IV (8). Reach and retrieve</u>

SET UP	TASK	VERBAL INSTRUCTIONS
<u>Starting Position:</u><br><br>• Chair Position (Front-Close).<br>• Hips against back of chair.<br>• Re-powder table if needed.<br>• 1 lb. weight centered on table and positioned just beyond 40-cm. line.<br>• Hand not being tested in lap.<br>• Elbow of arm to be tested extended, forearm in mid-position of pronation and supination and palm of hand in contact with weight.<br>• The subject must be able to maintain the starting position while the tester states "ready, set, go."<br>• Filming Position (Side).	<u>Task Description:</u><br><br>Patient attempts to pull 1 lb. weight across the 8-cm. line. Task object is a cuff weight folded so that it is approximately 7.6-cm. (3") on each side, and kept in place by a velcro fastener.<br><br><u>Timing Procedure:</u><br><br>Starts on the word "Go" and ends when any part of the weight crosses the 8-cm. line.<br><br><u>Measure:</u><br><br>The amount of time elapsed from the starting point to the moment the leading edge of the weight crosses the 8-cm line.	<u>Verbal Instructions:</u><br><br>• "Slide the weight across the table toward you until it is past the line nearest you. Do the task entirely by bending your elbow (demonstrate). The weight should remain in contact with your hand until it crosses the line. Do this as quickly as you can." (repeat instructions)<br>• "Do you have any questions?"<br>• "Ready, set, go."<br><br><u>Scoring:</u><br><br>• FA scoring should take into account: 1) the extent to which the head and trunk are maintained in normal alignment, 2) whether the activity is performed by bending the elbow as opposed to using excessive upper arm or hand movements (i.e., swatting the weight with the hand), and 3) the speed, fluidity, and precision with which movements are performed.<br>• If the patient's forearm loses contact with the weight or pronates, a maximum FA score of 3 should be assigned.<br>• If the patient is unable to maintain the starting position without physical assistance, a zero is assigned and activity is not attempted.

V. *Nine Tasks (VA-I – Tasks 9-17).* Functional ability of the arm and hand of the involved upper extremity; performed to the front of the patient.

VA(9). Lift can

**SET UP**	**TASK**	**VERBAL INSTRUCTIONS**
Starting Position:<br><br>• Chair Position (Front).<br>• Hips against back of chair.<br>• Hands in lap.<br>• Unopened 12-oz. soft drink can (392 gm) placed on table at patient's midline with front edge of can just beyond 20-cm. line.<br>• Filming Position (Side-Close).	Task Description:<br><br>Patient attempts to lift the can and bring it close to lips with a cylindrical grasp. An overhand grasp is not allowed for this task. Note - If the patient performs the task by lifting the can using an overhand grasp, repeat the task one more time. Assign a 120+ if the task cannot be accomplished in the correct manner.<br><br>Timing Procedure:<br><br>Starts on the word "Go" and ends when can is within approximately one inch of patient's mouth.<br><br>Measure:<br><br>The time elapsed from starting point to the moment the can comes within approximately one inch of patient's mouth.	Verbal Instructions:<br><br>• "Lift the can to your mouth without touching your lips, like this (demonstrate). It is important that you use the appropriate grasp and an overhand grasp is not allowed (demonstrate both grasps). Do this as quickly as you can." (repeat instructions)<br>• "Do you have any questions?"<br>• "Ready, set, go."<br><br>Scoring:<br><br>• FA scoring should take into account: 1) the extent to which the head and trunk are maintained in normal alignment, 2) whether the appropriate grasp is used (cylindrical grasp), 3) the directness of the trajectory to the mouth, and 4) the speed, fluidity, and precision with which movements are performed.

VB(10). Lift pencil

SET UP	TASK	VERBAL INSTRUCTIONS
Starting Position:<br><br>• Chair Position (Front).<br>• Hips against back of chair.<br>• Hands in lap.<br>• 7" pencil (with 6 flat sides) placed parallel to front edge of table, centered on patient's midline and with front edge of pencil at 20-cm. line.<br>• Filming Position (Side-Close).	Task Description:<br><br>Patient attempts to pick up the pencil using 3-jaw chuck grasp (thumb and first two fingers). The pencil should be picked up on the table and not over the edge of the table. Note - If the patient performs the task by lifting the pencil over the edge of the table once, repeat the task one more time. Assign a 120+ if the task cannot be accomplished in the correct manner.<br><br>Timing Procedure:<br><br>Starts on the word "Go" and ends when entire pencil (all surfaces) is raised from table at least ½ inch.<br><br>Measure:<br><br>The time elapsed from the starting point to the moment the entire pencil is raised from the table.	Verbal Instructions:<br><br>• "Pick up the pencil using your thumb and first two fingers and hold it in the air like this (demonstrate). The pencil should be picked up on the table and not over the edge of the table. Do this as quickly as possible."(repeat instructions)<br>• "Do you have any questions?"<br>• "Ready, set, go."<br><br>Scoring:<br><br>• FA scoring should take into account: 1) the extent to which the head and trunk are maintained in normal alignment, 2) whether the appropriate grasp is used (3-jaw chuck grasp), and 3) the speed, fluidity, and precision with which movements are performed.<br>• A 3-jaw chuck grasp should be used. If another grasp is used, a maximum FA score of 2 should be assigned.<br>• Raters should take into account the patient's control of the grasp. If the patient immediately drops the pencil, a maximum FA score of 3 should be assigned.

VC(11). Lift paper clip

SET UP	TASK	VERBAL INSTRUCTIONS
Starting Position:<br><br>• Chair Position (Front).<br>• Hips against back of chair.<br>• Hands in lap.<br>• 2" paper clip (coated and colored) placed parallel to the edge of table, centered on patient's midline, and with front edge of clip at 20-cm. line; the wider end of the paper clip should be facing towards the side to be tested.<br>• Filming Position (Side-Close).	Task Description:<br><br>Patient attempts to pick up the paper clip using a pincer grasp (pads of thumb and index finger opposed). The paper clip should be picked up on the table and not over the edge of the table. Note - If the patient performs the task by lifting the paper clip over the edge of the table once, repeat the task one more time. Assign a 120+ if the task cannot be accomplished in the correct manner.<br><br>Timing Procedure:<br><br>Starts on the word "Go" and ends when entire paper clip is off the table at least ½ inch.<br><br>Measure:<br><br>The time elapsed from the starting point to the moment the entire paper clip is raised from the table.	Verbal Instructions:<br><br>• "Pick up the paper clip using your thumb and index finger and hold it in the air like this (demonstrate). The paper clip should be picked up on the table and not over the edge of the table. Do this as quickly as possible." (repeat instructions)<br>• "Do you have any questions?"<br>• " Ready, set, go."<br><br>Special Consideration:<br><br>Fingernail length can significantly affect performance; therefore, patient should be instructed during the phone call making test arrangements to not clip fingernails for at least three days before test session.<br><br>Scoring:<br>• FA scoring should take into account: 1) the extent to which the head and trunk are maintained in normal alignment, 2) whether the appropriate grasp is used (pincer grasp), and 3) the speed, fluidity, and precision with which movements are performed.<br>• A pincer grasp should be used. If another grasp is used, a maximum FA score of 2 should be assigned.<br>• Raters should take into account the patient's control of the grasp. If the patient immediately drops the pencil, a maximum FA score of 3 should be assigned.

VD(12). Stack checkers

SET UP	TASK	VERBAL INSTRUCTIONS
Starting Position:<br><br>• Chair Position (Front).<br>• Hips against back of chair.<br>• Hands in lap.<br>• Three checkers are placed in a line parallel to front edge of table with front edge of each checker just beyond 20-cm. line. Checkers are spaced 4.5-cm. apart with middle checker at patient's midline.<br>• Filming Position (Side-close).	Task Description:<br><br>Patient attempts to stack the two end checkers onto the center checker. The task can be executed by picking up either checker first.<br><br>Timing Procedure:<br><br>Starts on the word "Go" and ends when patient has placed the third checker in required position.<br><br>Measure:<br><br>The time elapsed from the starting point to the moment the third checker is in place.	Verbal Instructions:<br><br>• "Stack the two end checkers onto the center checker. The checkers do not have to be perfectly stacked, but the top two checkers must not touch the table surface (demonstrate – note special consideration). Do this as quickly as you can."(repeat instructions)<br>• "Do you have any questions?"<br>• " Ready, set, go."<br><br>Special Considerations:<br><br>Checkers may be out of alignment, but in order for the task to be considered completed, the top two checkers may not be touching the table surface. The tester should demonstrate what is not acceptable.<br><br>Scoring:<br><br>• FA scoring should take into account the extent to which the head and trunk are maintained in normal alignment and the speed, fluidity, and precision with which movements are performed.<br>• The checkers do not need to be perfectly aligned; therefore, do not deduct rating points based on alignment of checkers.

VE(13). Flip cards

SET UP	TASK	VERBAL INSTRUCTIONS
Starting Position:<br><br>• Chair Position (Front).<br>• Hips against back of chair.<br>• Hands in lap.<br>• Three 3"x5" index cards placed in a line parallel to front edge of table, with short (3") far edge of card facing patient just beyond 20-cm. line. Cards spaced 3-cm. apart with middle card at patient's midline.<br>• Filming Position (Side-Close).	Task Description:<br><br>Using a pincer grasp on the near edge of cards, patient attempts to flip each of the cards over. This task should be done by sliding the front edge of the card just past the front edge of the table with some or all of the fingers and then grasping the card edge protruding past the table edge between the palmar surfaces of thumb and index finger. Cards should be flipped over from side to side (rather than from front to end). The cards do not have to be straightened or adjusted after they have been turned over. The patient should first flip over the card on side being tested, then the center card, and then the card on the opposite side. Patient should be prevented from wetting fingers by licking (which they commonly try to do).<br><br>Timing Procedure:<br><br>Starts on the word "Go" and ends when patient has flipped all cards into a new position.<br><br>Measure:<br><br>The time elapsed from the starting point to the moment the third card has been flipped over and released onto the table.	Verbal Instructions:<br><br>• "Flip each of the cards over. You should slide the card toward you so that it goes a little over the edge of the table. Start with the card on your (state side being tested) side, then the center card, and then the card on your (state opposite side) side. The cards should be flipped over from side to side rather than from end to end (demonstrate both correct and incorrect methods). The cards may land anywhere on the table, so you do not need to straighten the cards after turning them over. Do not lick your fingers and do the task as quickly as you can." (repeat instructions)<br>• "Do you have any questions?"<br>• "Ready, set, go."<br><br>Scoring:<br><br>• FA scoring should take into account: 1) the extent to which the head and trunk are maintained in normal alignment, 2) whether the forearm supinates when turning the cards, 3) the dexterity of the fingers, and 4) the speed, fluidity, and precision with which movements are performed.<br>• If the patient makes more than 2 attempts on any card, a maximum FA score of 2 should be assigned.<br>• If the patient fails to flip all cards side to side, a maximum FA score of 3 should be assigned.

VF(14). Grip strength

SET UP	TASK	VERBAL INSTRUCTIONS
Starting Position:<br><br>• Chair Position (Front-Close).<br>• Hand not to be tested on thigh.<br>• Hips against back of the chair.<br>• Upper extremity to be tested placed on table, olecranon process at front edge of table, forearm in neutral position, elbow flexed, shoulder slightly flexed and in 0^0 abduction.<br>• The hand-held dynamometer is set on the second setting position.<br>• Grip strength dynamometer placed in hand that is resting on the table. The tester or an assistant should stabilize the dynamometer for the patient from the front of the patient.<br>• Not Filmed.	Task Description:<br><br>Patient attempts to grip the dynamometer with greatest grip strength possible. The test should be conducted 3 times with a 1- minute rest between trials.<br><br>Timing Procedure:<br><br>Not applicable.<br><br>Measure:<br><br>The mean of grip strength exerted (kg) on 3 trials.	Verbal Instructions:<br><br>• "Squeeze the handle down as far as you can for at least 3 seconds and then let go when I say “Release.” I will ask you to do this 3 times with a 1-minute rest between attempts." (repeat instructions)<br>• "Do you have any questions?"<br>• " Ready, set, go."

VG(15). Turning key In lock

SET UP	TASK	VERBAL INSTRUCTIONS
Starting Position:<br><br>• Chair Position (Front).<br>• Hands placed on thighs.<br>• Hips against back of chair.<br>• Lock and key board is stabilized at a 45 degree angle, preventing board from moving when used by patient; board held parallel to front edge of table, just beyond 8-cm. line and centered on patient's midline.<br>• Filming Position (Side-Close)	Task Description:<br><br>Using a lateral pincer grasp, patient attempts to move the key in the lock from the vertical position first to the side being tested, then to the opposite side and finally back to the vertical starting position. Tumblers of the lock are set so that the key moves through a 180-degree arc (only), with 90 degrees of that arc on either side of the midline.<br><br>Timing Procedure:<br><br>Start on the word "Go". End when the key is in the starting position again.<br><br>Measure:<br><br>The time elapsed from the starting point to the moment the key is returned to the starting position.	Verbal Instructions:<br><br>• "Grasp the key between your thumb and your forefinger (demonstrate) and turn the key, first to the (state side being tested) as far as the key will turn, then to the (state the opposite side) as far as the key will turn and finally return the key to the original vertical position. There is a stop on either side. Be sure you move the key until you reach this point. Do this as quickly as you can." (repeat instructions)<br>• "Do you have any questions?"<br>• "Ready, set, go."<br><br>Scoring:<br><br>• FA scoring should take into account: 1) the extent to which the head and trunk are maintained in normal alignment, 2) whether the appropriate grasp is used (a lateral pincher grip), 3) whether the forearm moves into pronation and supination as the key is turned, and 4) the speed, fluidity, and precision with which movements are performed.<br>• If the patient doesn't tun the key in the correct sequence (i.e., turn the key to side being tested first), a maximum of 3 should be assigned for FA score.<br>• If a grasp other than a lateral pincer grasp is used, a maximum FA score of 3 should be assigned.

VH(16). Fold towel

SET UP	TASK	VERBAL INSTRUCTIONS
Starting Position:<br><br>• Chair Position (Front).<br>• Hands placed on thighs.<br>• Hips against back of chair.<br>• Face towel is placed flat on table centered on patient with front long edge of towel at 8-cm.line.<br>• Filming Position (Side-Close)	Task Description:<br><br>Patient picks up the towel with both hands, grasping the far corners of the towel. The patient first folds the towel lengthwise. The patient then folds the towel in half again across its center (widthwise). The second fold is done with the arm being tested only and is done from the side of the towel corresponding to the arm being tested. The folding does not need to be exact, but ends of the towel need to be approximately aligned (within 1.5 inches).<br><br>Timing Procedure:<br><br>Starts on the word "Go" and ends when the towel is completely folded on the table.<br><br>Measure:<br><br>The time elapsed from the starting point to the moment the towel is completely folded on the table.	Verbal Instructions:<br><br>• "Grasp the far corners of the towel and fold it lengthwise like this (demonstrate). Then fold it in half across its center by using your (state the side being tested) arm. Try to get the ends of the towel close together (demonstrate). In order to complete the task, the ends of the towel must be close together. Do the task as rapidly as possible. (repeat instructions)<br>• "Do you have any questions?"<br>• "Ready, set, go."<br><br>Scoring:<br><br>• FA scoring should take into account: 1) the extent to which the head and trunk are maintained in normal alignment, 2) the symmetry of the arms as they fold the towel for the first fold, and 3) the speed, fluidity, and precision with which movements are performed.<br>• The ends of the towel do not need to be exactly aligned after the second fold, but ends of the towel need to be approximately aligned (within 1.5 inches).

VI(17). Lift basket

SET UP	TASK	VERBAL INSTRUCTIONS
Starting Position:<br><br>• Patient standing and facing table.<br>• Bedside table (44" high) placed over the desk (29" high) on patient's side to be tested. The bedside table extends along the width of the desk.<br>• Basket at 8-cm. line on the test table template, leading edge 14-cm. from side edge of table of side to be tested, handles (taped together) lined up with center of body.<br>• Three-pound weight placed in basket.<br>• Filming Position (Front).	Task Description:<br><br>Patient attempts to pick up basket by grasping handle (from underneath the handle) and placing the basket on far edge of the rolling bedside table. The far edge of the basket should touch the far edge of the table.<br><br>Timing Procedure:<br><br>Starts on the word "Go" and ends when any portion of the base of the basket extends beyond the far edge of the bedside table.<br><br>Measure:<br><br>The time elapsed from the starting point to the moment the basket has been placed on the bedside table with any portion of the base of the basket beyond the far edge of the bedside table. (Note: release of the basket in not included in the time measure).	Verbal Instructions:<br><br>• "Pick up the basket with your (state the side being tested) hand and place the basket on the rolling table. The far edge of the basket should go past the far edge of the bedside table (demonstrate). Try not to move your feet while you do this task. Do this as quickly as you can." (repeat instructions)<br>• "Do you have any questions?"<br>• "Ready, set, go."<br><br>Scoring:<br><br>• FA scoring should take into account the extent to which the head and trunk are maintained in normal alignment and the speed, fluidity, and precision with which movements are performed.<br>• If the patient moves out of the original foot position, a maximum FA score of 3 should be assigned.<br>• The task is demonstrated with the leading edge of the basket crossing the far edge of the bedside table first. If other portions of the basket cross the far edge first, a maximum FA score of 3 should be assigned.<br>• The task is demonstrated without rotating the trunk. If the patient significantly rotates their trunk during the task, a maximum FA score of 3 should be assigned.

WOLF MOTOR FUNCTION TEST
DATA COLLECTION FORM

Subject's Name: ____ Date: _____________________

Test (check one): Pre-treatment _____ Post-treatment _____ Follow-up ____

Arm tested (check one): More-affected ______ Less-affected _____

	Task	Time	Functional Ability	Comment
1.	Forearm to table (side)		0 1 2 3 4 5	
2.	Forearm to box (side)		0 1 2 3 4 5	
3.	Extend elbow (side)		0 1 2 3 4 5	
4.	Extend elbow (weight)		0 1 2 3 4 5	
5.	Hand to table (front)		0 1 2 3 4 5	
6.	Hand to box (front)		0 1 2 3 4 5	
7.	Weight to box	______________________________lbs.		
8.	Reach and retrieve		0 1 2 3 4 5	
9.	Lift can		0 1 2 3 4 5	
10.	Lift pencil		0 1 2 3 4 5	
11.	Lift paper clip		0 1 2 3 4 5	
12.	Stack checkers		0 1 2 3 4 5	
13.	Flip cards		0 1 2 3 4 5	
14.	Grip strength	______________________________kgs.		
15.	Turn key in lock		0 1 2 3 4 5	
16.	Fold towel		0 1 2 3 4 5	
17.	Lift basket		0 1 2 3 4 5	

WMFT
PRE-TREATMENT SET-UP RECORDING FORM

Subject's name ______________________________ Date _____________________

Item:

1. Forearm to table:
   (check one): _____ standard _____ other (explain):
   ____
   ____

2. Forearm to box: Box size:_____
   (check one): _____ standard _____ other (explain):
   ____
   ____

3. Extend elbow (to the side):
   (check one): _____ standard _____ other (explain):
   ____
   ____

4. Extend elbow (weight):
   (check one): _____ standard _____ other (explain):
   ____
   ____

5. Hand to table (front):
   (check one): ____ standard _____ other (explain):
   ____
   ____

6. Hand to box (front): Box size:_____
   (check one): _____ standard _____ other (explain):
   ____
   ____

7. Weight to box (record sequence followed with weights): ____________________________________lbs.
   (check one) _____ standard _____ other (explain):
   ____
   ____

8. Reach and retrieve:
   (check one): _____ standard _____ other (explain):
   ____
   ____

9. Lift can:
   (check one): _____ standard _____ other (explain):
   ____
   ____

10. Lift pencil:

(check one): _____ standard _____ other (explain):
____
____

11. Lift paper clip:
(check one): _____ standard _____ other (explain):
____
____

12. Stack checkers:
(check one): _____ standard _____ other (explain):
____
____

13. Flip cards:
(check one): _____ standard _____ other (explain):
____
____

14. Grip strength: Trial 1:______kgs. Trial 2:_______kgs. Trial 3:_______kgs.
(check one): _____ standard _____ other (explain):
____
____

15. Turn key in lock:
(check one): _____ standard _____ other (explain):
____
____

16. Fold towel:
(check one): _____ standard _____ other (explain):
____
____

17. Lift basket
(check one): _____ standard _____ other (explain):
____
____

LIST OF TEST OBJECTS AND RELATED ITEMS FOR THE WMFT

The test objects and related items for the WMFT are listed below in the order in which they are used. Tasks should be administered with the subject seated in a standard chair at an appropriately sized desk or table (except for task 17 – done in standing). A laminated template should be secured to the tabletop or desktop. The template should be taped flush to the front edge of the table/desk and can be removed after testing.

Test objects

1. Box (cardboard) – 10-inches (25.4 cm.) in height. This represents approximate shoulder height for the average adult. An 8-inch (20.3 cm.) and 6-inch (15.2 cm.) box should also be available for shorter individuals.
2. 1 lb. Cuff-weight with Velcro strap.
3. 1-20 lb. Cuff-weight with removable weight inserts.
4. Unopened 12-oz. soft drink can (392 gm.).
5. 7-inch (17.78 cm.) pencil with 6 flat sides.
6. 2-inch (5.08 cm.) paper clip (colored and coated with plastic).
7. Three standard checkers.
8. Three 3-inch (7.62 cm.) x 5-inch (12.7 cm.) index cards.
9. Standard grip strength dynamometer.
10. Lock and key secured to a board that is placed at a 45-degree angle. Tumblers are set so the key moves through a 180-degree arc (only), with 90 degrees of that arc on either side of midline.
11. Standard dish towel: 25-inch (63.5 cm.) x 15-inch (38.10 cm.).
12. Plastic or wicker tote basket with handle – approximately 15 inches (38.1 cm.) in height, 8.5 inches (21.6 cm.) in width, and 14 inches (35.56 cm.) in length.

Related items

1. Desk/table of standard height – approximately 29-inches (73.5 cm.) in height, 54-inches (137cm.) in width, and 30-inches (76 cm.) in length.
2. Straight back chair – seat 18-inches (45.7 cm) high, without armrests.
3. Template to be taped flush to the desk/table top to indicate test object placement.
4. Talcum/baby powder.
5. Stopwatch.

Functional Ability Scale

0 – Does not attempt with upper extremity (UE) being tested.

1 –UE being tested does not participate functionally; however, attempt is made to use the UE. In unilateral tasks the UE not being tested may be used to move the UE being tested.

2 – Does, but requires assistance of the UE not being tested for minor readjustments or change of position, or requires more than two attempts to complete, or accomplishes very slowly. In bilateral tasks the UE being tested may serve only as a helper.

3 – Does, but movement is influenced to some degree by synergy or is performed slowly or with effort.

4 – Does; movement is close to normal *, but slightly slower; may lack precision, fine coordination or fluidity.

5 – Does; movement appears to be normal *.

(*) For the determination of normal, the less-involved UE can be utilized as an available index for comparison, with pre-morbid UE dominance taken into consideration.

Adapted from © 2011 UAB CI Therapy Research Group
