# Supplementary material for: Effects of combining two techniques of non-invasive brain stimulation in subacute stroke patients: a pilot study
Source: BMC Neurol. 2022 Mar 17;22:98. doi: 10.1186/s12883-022-02607-3 (PMC8928603; doi:10.1186/s12883-022-02607-3)
Supplement: Supplementary file 7 — Additional file 7. Subgroup analysis with adjusted baseline. Supplementary 1. Baseline-difference values for timepoints 1 (T1) and 2 (T2). Supplementary 2. The 2-way mixed ANOVA of FMA-UE with adjusted baseline. Supplementary 3. Post-hoc analysis using Bonferroni adjustment. [file 12883_2022_2607_MOESM7_ESM.pdf]

Additional file 7_Subgroup analysis with adjusted baseline

Supplementary 1. Baseline-difference values for timepoints 1 (T1) and 2 (T2)

General Linear Model

Within-Subjects Factors

Measure: MEASURE_1

fma	Dependent Variable
1	dif12
2	dif13

fma 1 = active; fma 2 = sham; dif12 = T1-T0; dif 13 = T2-T0

Descriptive Statistics

	1	Mean	Std. Deviation	N
dif12	1	1.6000	2.19089	5
	2	.6000	1.34164	5
	Total	1.1000	1.79196	10
dif13	1	9.4000	3.64692	5
	2	1.8000	2.04939	5
	Total	5.6000	4.88080	10

dif12 = T1-T0; dif 13 = T2-T0; 1-1 =active; 1-2 = sham

Comment

We adjusted the baseline by subtracting T0 from T1 (dif12) and T0 from T2 (dif13). Data distribution is shown as a mean and standard deviation, as in descriptive statistics.

Supplementary 2. The 2-way mixed ANOVA of FMA-UE with adjusted baseline

Tests of Within-Subjects Effects

Measure: MEASURE_1

Source		Type III Sum of Squares	df	Mean Square	F	Sig.
fma	Sphericity Assumed	101.250	1	101.250	28.125	.001
	Greenhouse-Geisser	101.250	1.000	101.250	28.125	.001
	Huynh-Feldt	101.250	1.000	101.250	28.125	.001
	Lower-bound	101.250	1.000	101.250	28.125	.001
fma * group	Sphericity Assumed	54.450	1	54.450	15.125	.005
	Greenhouse-Geisser	54.450	1.000	54.450	15.125	.005
	Huynh-Feldt	54.450	1.000	54.450	15.125	.005
	Lower-bound	54.450	1.000	54.450	15.125	.005
Error(fma)	Sphericity Assumed	28.800	8	3.600		
	Greenhouse-Geisser	28.800	8.000	3.600		
	Huynh-Feldt	28.800	8.000	3.600		
	Lower-bound	28.800	8.000	3.600		

Comment

The overall FMA-UE motor score was statistically significant across two-time points, F (1, 8) = 28.125, p = 0.001, and there was a significant interaction between time and group of experiments, F (1, 8) = 15.125, p = 0.005, implying that the change in scores over time differed between the groups assigned even after the baseline was adjusted.

Supplementary 3. Post-hoc analysis using Bonferroni adjustment

Pairwise Comparisons

Measure: MEASURE_1

fma	(I) 1	(J) 1	Mean Difference (I-J)	Std. Error	Sig.[b]	95% Confidence Interval for Difference[b] Lower Bound	Upper Bound
1	1	2	1.000	1.149	.409	-1.649	3.649
	2	1	-1.000	1.149	.409	-3.649	1.649
2	1	2	7.600*	1.871	.004	3.286	11.914
	2	1	-7.600*	1.871	.004	-11.914	-3.286

fma 1 = T1; fma 2 = T2;(I)1-1= active; (J)1-2 = sham

Comment

Pairwise comparisons between groups using Bonferroni's correction, found that FM-UE of the active group [(I)1-1] had a significantly higher motor score only at 1 week after stimulation (T2), p =.004, as compared to sham [(J)1-2] after baseline adjustment.
